# Malaria prevalence in Commune 5 in Tumaco (Nariño, Colombia)

**DOI:** 10.12688/f1000research.110361.3

**Published:** 2023-07-19

**Authors:** Pablo Enrique Chaparro Narváez, Monica Marcela Jimenez-Serna, Maria Luz Gunturiz Albarracin, Gabriel Carrasquilla Gutierrez

**Affiliations:** 1 Observatorio Nacional de Salud, Instituto Nacional de Salud, Bogotá, 111321, Colombia; 2 Fundación Santa Fe de Bogotá, Bogotá, 110111, Colombia; 3 Dirección de Investigación en Salud Pública, Instituto Nacional de Salud, Bogotá, 111321, Colombia; 4 Fundacion Santa Fe de Bogotá, Bogotá, 110111, Colombia

**Keywords:** prevalence, diagnosis, chromosome deletion, urban malaria, Colombia

## Abstract

Background

Urban malaria is a public health problem in Colombia and there is still lack of knowledge about its epidemiological characteristics, which are key to the implementation of control measures. The presence of urban malaria cases and disease diagnosis are some of the challenges faced by malaria elimination programs. The objective of this research was to estimate malaria prevalence, explore associated factors and detect *pfhrp* *2/3* genes, in the urban area of Tumaco between July and December 2019.

Methods

A prevalence study was conducted by using a stratified random probability sample. Structured surveys were administered and blood samples were taken and examined through optical microscopy, rapid diagnostic tests (RDT) and polymerase chain reaction (PCR). A logistic regression model was used to explore associated factors.

Results

1,504 people living in 526 households were surveyed. The overall prevalence was 2.97% (95% CI: 2.1 - 4.3%). It was higher in males, in the 10-19 age group and in asymptomatic cases. The prevalence of *pfhrp2* amplification was 2.16% (95% CI: 1.6 - 2.9%). Households with three or more people had a higher risk of malaria infection (adjusted odds ratio (ORa) 4.05; 95% confidence interval (CI) 1.57-10.43). All cases were due to *P.* *falciparum.*

Conclusions

The prevalence of urban malaria was low. Strategies to eliminate malaria in urban areas should be adjusted considering access to early diagnosis, asymptomatic infection, and the RDTs used to detect the presence of the *pfhrp2* gene.

## Introduction

Malaria elimination programs face several challenges, including urban malaria and disease diagnosis. Both in Colombia and worldwide, malaria transmission is mainly rural, but cases of malaria in urban and peri-urban areas have been continuously reported over the last decade, most of them in the Pacific region. ^1^^,^^2^ These problems are compounded by the diagnosis of asymptomatic and submicroscopic malaria.

Despite the fight against malaria, the World Health Organization (WHO) observed an increase in cases worldwide, increasing from 227 million in 2019 to 241 million in 2020 in the 85 countries where the disease is endemic. Similarly, in terms of mortality, 558,000 deaths were registered in 2019 and 627,000 in 2020. However, in the Americas region, the WHO reported a reduction in cases, from an estimated 893,000 in 2019 to 652,000 in 2020, 77% of them in Venezuela, Brazil and Colombia. Meanwhile, there was a reduction in deaths, from 509 in 2019 to 409 in 2020. ^3^

It is worth mentioning that 91% of the Colombian territory has eco-epidemiological conditions that promote malaria transmission and it is estimated that 22% of the population lives in these areas. In 2020, 81,363 cases were registered (annual parasite index (API) 8.4 cases per 1,000 inhabitants) and 5 deaths were confirmed in the country. The predominant parasites were *Plasmodium vivax* (49.7%) and *Plasmodium falciparum* (49.5%). ^4^ On the Pacific coast, in the department of Nariño, where Tumaco is located, 17,421 cases were reported (API 31.9 cases per 1,000 inhabitants). ^4^ According to the Vector-Borne Disease Prevention and Control Program, cases of urban malaria have been identified mainly in the neighborhoods that are part of Commune 5 in Tumaco, where 1,024 cases were reported in 2015 (API 42.2 per 1,000 inhabitants) and 770 in 2016 (API 31.7 per 1,000 inhabitants).

In Colombia, the strategic action route for comprehensive care, health promotion, prevention, surveillance, control and elimination of malaria has been based on the “2019-2022 National Malaria Strategic Plan” which, among other aspects, includes disease diagnosis through the use of optical microscopy and rapid diagnostic tests (RDTs), and the elimination of urban and peri-urban malaria in 18 municipalities on the Pacific coast. ^5^

Optical microscopy can detect from 50 to 100 parasites/μL but it can lead to diagnostic errors when parasitemia is low. ^6^ The purpose of RDTs is to detect one of three antigens in parasites: lactate dehydrogenase (LDH), aldolase, and histidine-rich protein 2 (hrp2), with significant differences from each other. ^7^ The detection limit for RDTs is between 100-200 parasites/μL. ^8^ It should be noted that *hrp2* is specific for *P. falciparum* and that *pfhrp2*-based RDTs often detect the *pfhrp3* antigen, rendering test interpretation difficult. ^9^ Test performance may be affected by the antigenic variability of the target protein, the persistence of the antigen in blood after parasite elimination, and parasite density below the detection threshold. The presence of parasites with deletions or mutations of the *pfhrp2* gene can lead to false-negative results, which has implications for RDT implementation, case diagnosis and treatment, and malaria control and elimination efforts. ^10^

To overcome the limitations of optical microscopy and RDTs, molecular detection of malaria parasites through the polymerase chain reaction (PCR) was used, as it can detect parasitemia below 0.05 μL. ^7^ On the other hand, PCR provides better information on the prevalence and distribution of parasitic species in endemic areas compared to that provided by optical microscopy and RDT. ^11^ PCR is a more sensitive diagnostic method to detect asymptomatic infections with very low parasite density. It may be useful in studies of submicroscopic infections, although its utility depends on the epidemiological importance of low-density infections, which have not yet been characterized. ^12^

Regarding urban malaria, there are challenges in terms of what is meant by “urban”, “peri-urban”, and “rural”, because of the lack of established definitions to describe the socioeconomic and ecological contexts where the disease is transmitted. ^1^^,^^13^ This situation is compounded by the failure to establish the origin of malaria cases, to identify mosquito breeding sites, and to confirm the vector's transmission capacity in these environments. ^1^ Despite these difficulties, it has been established that urban and peri-urban malaria may be transmitted by population traveling from rural to urban and peri-urban areas. ^14^^,^^15^ Local transmission has also been suggested in urban areas where the presence of *Anopheles*, as a competent vector, and infected people converge. ^14^ In Latin America, peri-urban transmission has been reported in Brazil (Porto Velho) ^16^ and Peru (Iquitos, Sullana, Piura y Lima). ^17^^–^^19^ In Colombia, urban malaria has been identified in municipalities on the Pacific coast, where Tumaco is located. Peri-urban malaria is caused by the displacement of rural populations to peri-urban areas as a result of the armed conflict, presence of paramilitary groups, illicit crops, and illegal mining. ^1^

Most studies conducted in Colombia have addressed the prevalence of malaria in rural areas. Research focused on urban malaria has been scarce, reporting on total prevalence and the prevalence of asymptomatic and submicroscopic infections. ^1^^,^^13^^,^^20^^–^^26^ Also, research aimed at detecting the *pfhrp 2/3* gene deletion has been limited (Guapi 6%). ^27^ Given the limited research on malaria prevalence carried out in the Colombian territory and the detection of *pfhrp 2/3* genes in urban areas, this study was aimed at estimating malaria prevalence, detecting *pfhrp 2/3* genes, and exploring associated factors in the urban area (Commune 5) in Tumaco during 2019.

## Methods

### Ethics and consent

This study was approved by the ethics committees of the National Institute of Health (April 4, 2017 Minutes) and Fundación Santa Fe de Bogotá (April 22, 2019 Minutes). Participants’ written consent was obtained prior to data collection and blood sampling. The research with mosquitoes considered the Colombian scientific, technical, and administrative standards for biomedical research with animals. The mosquitoes were anesthetized with triethylamine before dying.

### Study design

**Figure 1.**
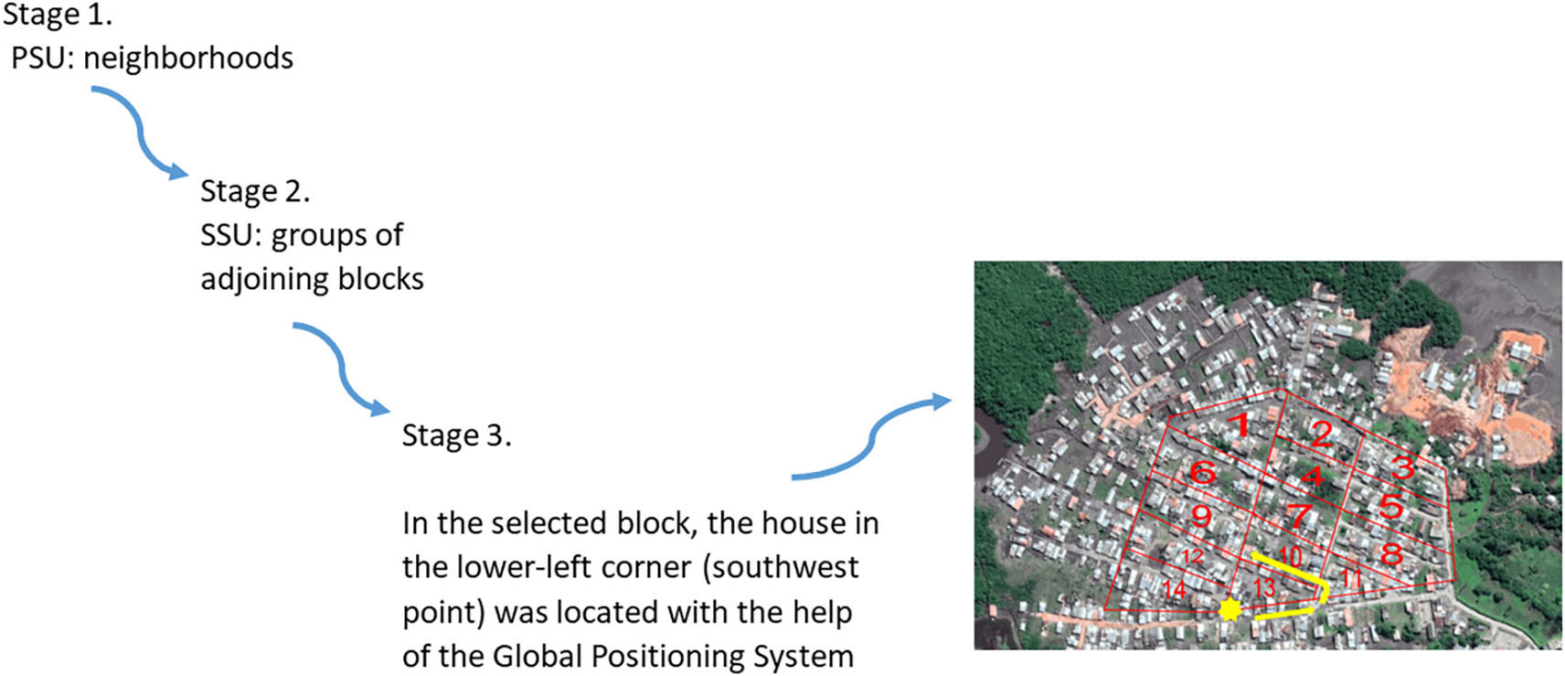
Diagram of the study sampling technique. PSU: primary sampling units, SSU: secondary sampling units.

Between July and December 2019, a prevalence study was conducted in Commune 5 of Tumaco, a malaria-endemic urban area. This Colombian municipality is divided into communes and, in turn, each commune is made up of neighborhoods. A stratified (by neighborhoods) multistage random sample with proportional allocation was established. In the first stage, the primary sampling units were the Commune 5 neighborhoods. In the second stage, the secondary sampling units were groups of adjoining blocks. A grid was placed on the map of each selected neighborhood. Each cell included the residential units that make up a block. Sampling was carried out proportional to size, taking into account the number of inhabitants per block. Using the Teaching Sampling package of the statistical package R, the blocks in each of the selected neighborhoods were chosen. In the third stage, in the selected block, the house in the lower-left corner (southwest point) was located with the help of the Global Positioning System (GPS). In this dwelling began the collection of information. Then the selection of the next dwelling to the right or to the left depended on the outcome of a coin toss. Once the address was established, the collection of information continued until completing 12 contiguous houses. The units of analysis and observation were all individuals in the selected households ( Figure 1).

To calculate sample size, a prevalence of 4.22% was considered, as reported by the “Vector-Borne Disease Prevention and Control Program in the Nariño Department” study in 2015. Accuracy was estimated to have a 15.0% expected relative standard error, ^28^ 95% confidence, and a 1.5 cluster design effect. Adjusting for a non-response rate of 10%, the final sample size was 1,424 individuals from households in Commune 5.

Officials from the departmental and municipal Vector-Borne Disease Prevention and Control Program, community leaders, and researchers informed the population of Commune 5 about the study. This ensured community participation in the study.

The inclusion criteria for the study were: (1) inhabitants of the selected dwelling who voluntarily wish to participate and who at the time of the visit were in the dwelling, (2) minors who have the authorization of the responsible adult or guardian, (3) people who have adequately filled out the informed consent. The exclusion criteria were: (1) people with mental problems unable to sign informed consent and (2) those for whom a blood sample was not possible to obtain.

### Study area

**Figure 2.**
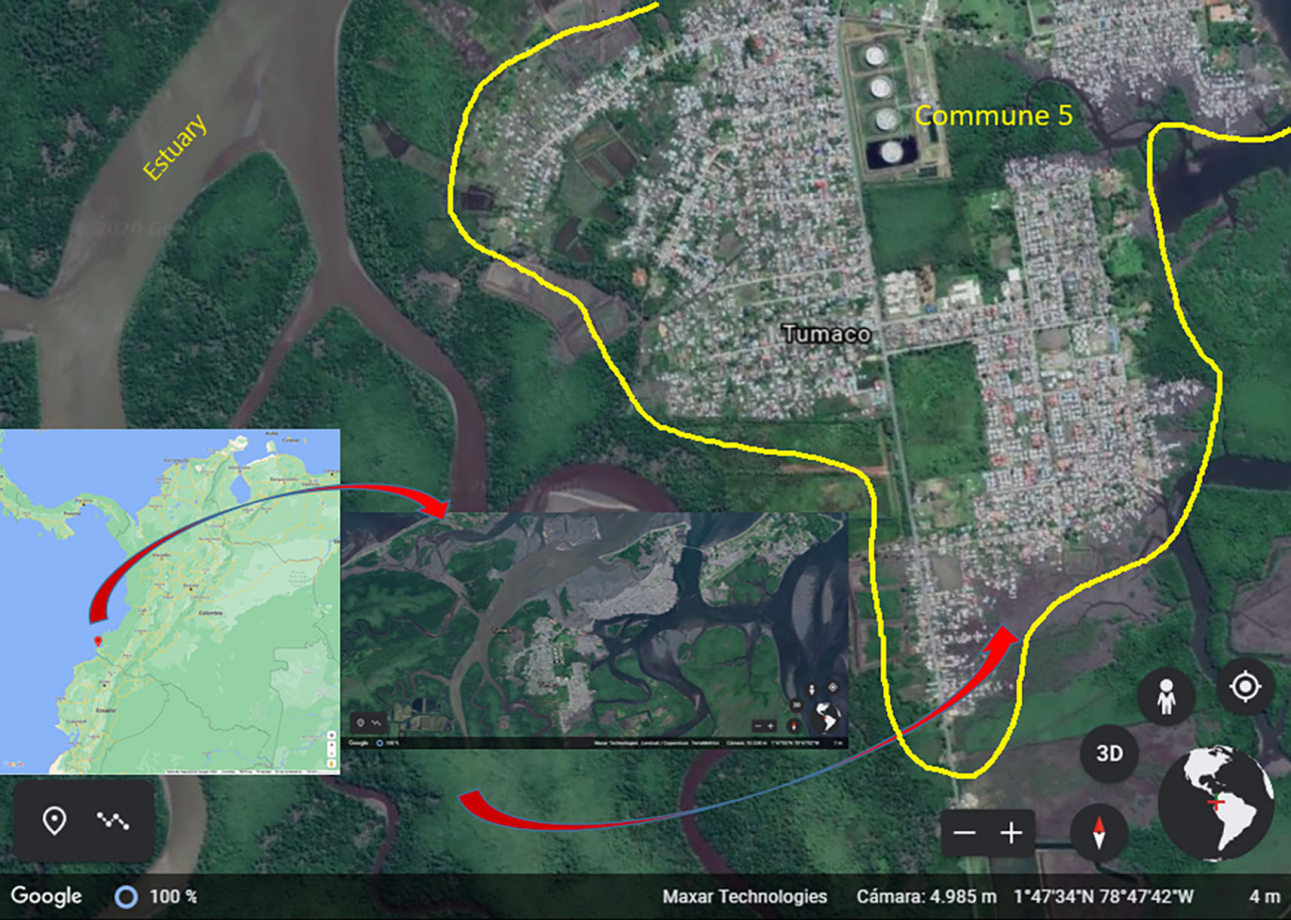
Study area in Tumaco (Nariño, Colombia). Sources: Google Maps: Maps Data: Google. © 2022 INEGI (retrieved on March 10. 2022). Google Earth: Maps Data: Google. © 2020 Maxar Technologies (retrieved on December 13. 2020).

Tumaco is located in southwestern Colombia (1° 48' 24” N; 78° 45' 53” W), on the Pacific coast, Nariño Department. It spreads over 3,778 km ^2^ and the urban area is about 38 km ^2^. This area is made up of three islands (El Morro, La Viciosa and Tumaco), which are divided into five communes and 82 neighborhoods ^29^ ( Figure 2). The municipality is located 2 meters above sea level and has a warm humid weather. It has an average annual temperature of 26.1°C, a relative humidity of 84.8%, and annual precipitation ranging from 109 to 373 mm ^3^. In 2021, its population was estimated at 257,042 inhabitants, out of which 33.7% lived in the urban area. ^30^ Its economy is based on the production of African palm, cocoa and coconut and, to a lesser extent, tourism. In the urban area of Tumaco, all five communes are endemic to malaria. In this city, 70% of malaria cases were reported from Commune 5 in the last ten years. Larval habitats correspond to wells, ponds, puddles, and lakes for shrimp farming. Commune 5, with 26,000 inhabitants, is located on the continental area of Tumaco on the low-tide land and nearby estuaries. The presence of *Anophelines spp.* breeding sites (ponds, puddles, and lakes for shrimp farming) and high density of the population make this commune of high risk of malaria transmission.

### Data collection

Two structured questionnaires were designed. The first one included variables related to households characteristics, such as basic services and the presence and use of long-lasting insecticide-treated nets. The second one included sociodemographic variables (age, sex, ethnicity, marital status, occupation, educational level, socioeconomic stratum, belonging to the General Social Security Health System), symptoms, diagnosis and history of malaria. The questionnaires, created on mobile data capture devices (Android phones), were applied face-to-face by previously trained personnel. Interviews were conducted in Spanish. At the end of each study day, data collected were reviewed by the study coordinator, and any quality issues were flagged for immediate correction.

### Laboratory procedures

*Optical microscopy*

Blood samples were taken through finger puncture with a sterile lancet and each sample was then put on a clean film. Blood samples was taken at the household and thick smear was taken to the “Vector-Borne Disease” (VBD) control program and read by a bacteriologist according to national guidelines. ^31^ Parasitemia was estimated based on a 200-leukocyte count (assuming a standard value of 8,000 leukocytes/μL of blood) and was expressed as parasites/μL (p/μL). The National Institute of Health performed quality control on 10% (153) of the samples. All participants with positive thick blood film received antimalarial treatment according to national guidelines.

*Rapid diagnostic tests (RDTs)*

For RDTs, a second drop of blood was obtained from the same finger puncture and it was then put in the RDT device, according to the manufacturer's instructions. The RDT used (SD BIOLINE Malaria Ag P.f/P.v) was the one distributed by the National VBD Prevention and Control Program to all municipalities in the country. The same bacteriologists who performed the optical microscopy diagnosis analyzed the RDTs at the household. The National Institute of Health performed quality control on 10% (153) of the RDT devices used.

*Polymerase chain reaction (PCR)*

For PCR, two drops of blood were obtained from the same finger puncture and were then put on the filter paper. 10 mm 2 of Whatman #3 filter paper impregnated with blood were taken and genomic DNA extraction was performed according to the protocol described below. This procedure was performed in duplicate.

*Standardization of protocols for genomic DNA extraction from filter paper samples*

The saponin/chelex-100 method was used for DNA extraction. Briefly, a quarter of each dry droplet on filter paper was cut into small pieces and incubated at room temperature with a 0.5% saponin solution diluted in Phosphate buffered saline PBS. Then, the solution was mixed using a vortex and the supernatant was discarded. A wash was done with PBS, and 200 ul of a Chelex-100 solution diluted in water was added, then it was incubated at 56°C for 1 hour and at 100°C for 20 minutes. After incubation, the supernatant was transferred to sterile Eppendorf tubes and stored at -20°C until it was going to be used in the PCRs. gDNA was quantified by using a Nanodrop 2000, obtaining quality between 1.8 and 2.0. Once the gDNA had been extracted, diagnosis was performed through conventional PCR for the amplification of the gene fragments coding for the *pfhrp2* and *pfhrp3* proteins and the gene coding for the small subunit of *Plasmodium vivax*-PvSSU ribosomal RNA. DNA from reference strains was used as positive control. Negative controls without DNA and extraction controls obtained from filter paper without DNA were also included for each of the reactions. For the amplification of the different gene fragments mentioned before, specific primers were designed and PCR conditions were standardized for each of them. Guanine/cytosine (GC) content, dimer formation, loop formation, palindromes and melting temperature (MT) were taken into account for primer design. The sequences of the *hrp2/hrp3* and PvSSU genes published in Genbank were used, an alignment was performed by using clustalW (Refseq), and primers were designed from conserved regions of the sequences obtained.

PCR products were fractionated on 1.8% agarose gels stained with ethidium bromide, using a 100 bp molecular weight marker (Promega) as reference. The amplified products were visualized on a UV transilluminator and were purified on 1.8% agarose gels by using the GFX PCR DNA kit and Gel Band purification system (GE Healthcare). The purified products were sequenced by using the oligonucleotides used in the PCR, through the BigDye Terminator v3.1 cycle sequencing kit (Applied Biosystems) and the ABI PRISM 310 genetic analyzer. Sequence editing and analysis was performed through the DNA Sequencing Analysis software version 5.3.1. ^32^^,^^33^ Sequence comparisons were made by using the blastn tool available at the National Center for Biotechnology Information (NCBI).

For the amplification of the different fragments of the genes mentioned, we designed the specific primers and the PCR conditions for each of them were standardized. For the design of the primers, the GC content, dimer formation, loop formation, palindromes and Tm were taken into account. The sequences of the HRP2/HRP3 and PvSSU genes published in the Genbank were used, an alignment was performed using clustalW (Refseq) and the primers were designed from conserved regions of the sequences obtained.

For the PCRs, the Invitrogen™ DNA polymerase Taq recombinant kit was used (Includes: Taq DNA Polymerase (5 U/μL); 10× PCR buffer (200 mM Tris-HCl pH 8.4, 500 mM KCl); Magnesium Chloride (50 mM). Bio-Rad C1000 Thermal Cycle was used for PCRs. Glyceraldehyde-3-phosphate dehydrogenase (GAPDH) was used as housekeeping gene. The Table 1 shows the standardized amplification conditions for each gene, primers and expected fragment sizes.

**Table 1.** Standardized amplification conditions for each gene, primers and expected fragment sizes (PCR: polymerase chain reaction).

Gene name	Acronym gene	Primers	Expected product size	PCR amplification conditions
Gene that codes for histidine-rich protein 2 of *Plasmodium falciparum*	PfHRP2	**Pfhrp2-F** 5´-CAAAAggACTTAATTTAAATAAgAg-3´ **Pfhrp2-R** 5´-AATAAATTTAATggCgTAggCA-3´	882 bp	95°C/5 min 39 cycles: 95°C/1 min; 55°C/1 min; 72°C/1 min 72°C/7 min 12°C/∞
Gene that codes for histidine-rich protein 3 of *Plasmodium falciparum*	PfHRP3	**PfHRP3-F** 5′-AAATAAgAgATTATTACACgAAAg-′3 **PfHRP3-R** 5′-TGGTgTAAgTGATgCgTAGT-′3	615 bp	95°C/5 min 35 cycles: 95°C/1 min; 51°C/1 min; 72°C/1 min 72°C/7 min 12°C/∞
Gene that codes for the small ribosomal RNA subunit of *Plasmodium vivax*	PvSSU	**PvSSU-F** 5′-gTTAAgggAgTgAAgACgATC-′3 **PvSSU-R** 5′-AACCCAAAgACTTTgATTTCTCA-′3	159 bp	95°C/5 min 35 cycles: 95°C/1 min; 50°C/45 sec; 72°C/1 min 72°C/5 min 12°C/∞
Gene that codes for Glyceraldehyde-3-phosphate dehydrogenase	GAPDH	**GAPDH-F** 5′-ATggCAgTAACAAAACT-′3 **GAPDH-R** 5′-TgAATCgTATTTAAgAgA-′3	389 bp	95°C/5 min 35 cycles: 95°C/1 min; 53°C/30 sec; 72°C/1 min 72°C/7 min 12°C/∞

### Entomological study

The entomological study did not use a probabilistic sample. The mosquito collection took place in homes that were included in the prevalence study and whose residents agreed to participate. Between October 1 and 23, 2019, 81 capture points were sampled in 25 homes and 83 breeding places. Mosquitoes were collected through suction tubes and simultaneously with Centers for Disease Control and Prevention (CDC) and Shannon traps at rest (by the research group) and human bait (by the VBD staff) starting at 18:00 hours and ending at 6:00 hours in households in the Obrero neighborhood. Given the presence of illegal armed groups, collection in the Los Ángeles, Candamo, Unión Victoria and Ciudadela neighborhoods was authorized between 18:00 and 24:00 hours. From this time and until 6:00 hours, CDC traps were used. In the Once de Noviembre and Nuevo Milenio neighborhoods, permission was not obtained and mosquitoes were collected through traps that were installed between 18:00 and 6:00 hours. Mosquitoes were collected simultaneously inside and outside of selected households.

The collected mosquitoes were placed in containers labeled with date, neighborhood, house code, time of collection, number of mosquitoes and collector name. Specimens were killed with triethylamine and then individually packed in 1.5 ml vials with perforated caps. They were preserved in airtight bags with silica gel. Specimens of adult and immature mosquitoes were determined by using dichotomous keys for *Anopheles spp.* in Colombia. ^34^^–^^37^

Also, immature forms were searched in breeding sites within a radius of 1,000 meters around the selected households. Each breeding site was geo-referenced and its physical and environmental characteristics were registered. Sampling was carried out with a standard ladle, ten dips per square meter. Larvae were stored in 120 ml Whirl-Pak plastic bags with ethanol for preservation. Each larvae container was labeled with date, code, larva number, neighborhood and collector name.

### Definitions

This study assumed the definitions of symptomatic malaria, asymptomatic malaria, and submicroscopic malaria. Symptomatic malaria. Individual with positive microscopy/RDT and symptoms of malaria, such as fever, chills, vomiting, convulsions, malaise, headache and/or loss of appetite.

Asymptomatic malaria. Individual with positive microscopy/RDT or PCR, without symptoms. ^38^^,^^39^

Submicroscopic malaria. Individual with positive PCR and negative microscopy/RDT. Submicroscopic infections are almost exclusively asymptomatic. ^39^

### Statistical analysis

The data were obtained through mobile devices. After quality control, they were exported and analyzed with the Stata ^R^ version 12 statistical package. For statistical analysis, weights were generated according to strata, expansion factors and design effect, and the complex sample analysis module (svy command) was used. Categorical variables are presented as unweighted counts and weighted proportions with their corresponding 95% confidence intervals (95% CI), and continuous variables are expressed as means with 95% CI. The prevalence of malaria infection was estimated with the corresponding 95% CI. Malaria prevalence was calculated by age group, sex, symptoms and neighborhoods. The exploratory analysis of factors associated with urban malaria and households and urban malaria and individual was performed through logistic regression, first evaluating the relationship between each independent variable with the urban malaria variable. A p<0.05 value was considered statistically significant and those with p<0.05 were selected for the multivariate model.

## Results

### Housing and demographic characteristics

The total number of respondents was 1,504 people (60.0% female) residing in 526 households in the selected neighborhoods of Commune 5 in Tumaco. The average age of the respondents was 29.75 years (95% CI, 28.75 to 31.22).

Household characteristics are included in Table 2. More than half of the households' exterior walls were made of brick. More than two thirds had water service, while access to sewage service was scarce. Malaria was detected in 7.3% of households.

**Table 2.** Household characteristics. Tumaco, July to December 2019.

Characteristics	Total			
	Unweighted count	Weighted count		
	n	(%)	95% Confidence Interval	
			Lower	Upper
**Exterior wall material**				
Wattle and daub	40	7.7	5.1	11.3
Brick	294	56.1	48.6	63.3
Wood	156	29.1	23.6	35.3
Others	36	7.1	5.2	9.6
**The household has**				
Windows	484	92.5	88.6	95.1
Electricity	522	99.5	97.8	99.9
Gas	510	96.4	94.8	97.5
Water	405	72.5	63.9	79.7
Sewage	1	0.3	0	1.9
Garbage collection	479	91.5	85.8	95.1
**Number of people living in the household**				
1 and 2	276	52.6	48.3	56.9
3 or more	250	47.4	43.1	51.7
**Rooms where people sleep**				
1 and 2	301	57.3	52.1	62.4
3 or more	225	42.7	37.6	47.9
**Socioeconomic stratum**				
1	525	99.8	98.4	100
2	1	0.2	0	1.6
**Inhabited days per week**				
7 days	522	99.1	96.1	99.8
<7 days	3	0.9	0.2	3.9
**Households where people use a protective net while sleeping**	515	98.0	95.9	99.0
**Households with malaria cases**	35	7.3	5	10.5

Respondents' characteristics are shown in Table 3. There was higher participation of women and individuals between 20 and 49 years old. The majority were of African descent (95.2%). Nearly half of the individuals had lived in the households for over ten years (47%). Two-fifths of the respondents had high school education. One-third were workers and one-fourth were engaged in household activities. The family income for four-fifths of the participants was less than the minimum wage for one person. In the last year, 0.67% of the participants reported they had had malaria.

**Table 3.** Characteristics of the surveyed population. Tumaco, July to December 2019.

Characteristics	Total			
	Unweighted count	Weighted count		
	n	%	95% Confidence Interval	
**Age groups**				
0-9	272	18.32	16.0	20.9
10-19	318	21.47	19.1	24.1
20-49	626	40.88	38.64	43.15
≥50	288	19.33	16.94	21.98
**Sex**				
Male	531	35.56	33.5	37.7
Female	973	64.44	62.3	66.5
**Ethnic group**				
Black, African descent	1,439	95.23	91.4	97.4
Others	65	4.77	2.6	8.6
**Years living in the house**				
0 to 4	373	25.60	22.5	28.9
5 to 9	397	25.97	22.4	29.9
10 or more	714	47.03	42.6	51.5
No information	20	1.40	0.8	2.3
Educational level ^*^				
Preschool, primary	555	40.75	37.05	44.55
High school	628	45.79	42.88	48.73
University	113	8.54	6.43	11.24
None	72	4.93	3.57	6.77
Civil status ^**^				
Has a partner	585	52.78	49.08	56.45
No partner	526	47.22	43.55	50.92
**Occupation**				
Worker	450	30.04	26.4	34.0
Housework	399	26.26	24.0	28.7
Early childhood	158	10.46	8.8	12.4
Childhood	178	12.26	10.7	14.0
Adolescence	218	14.49	12.7	16.5
Unemployed	36	2.29	1.6	3.2
Pensioner	14	0.86	0.5	1.4
Other	51	3.34	2.4	4.6
**Family income**				
<1 MLW (Monthly Legal Wage)	1,219	79.37	72.8	84.7
1 or more MLW	281	20.33	15.1	26.8
Does not know	4	0.30	0.1	1.7
**Malaria in the last year**	10	0.67	0.32	1.40

^*^ In the “Educational level” variable, 136 children under 5 years of age were excluded.

^**^ In the “Civil status” variable, 393 children under 14 years of age were excluded.

### Prevalence of malaria

The prevalence was established from 1,504 microscopic examinations, 1,504 rapid diagnostic tests, and 1,504 PCR.

The total prevalence of malaria was 2.97% (95% CI: 2.1-4.3%). The prevalence obtained through microscopy/RDT was 0.81%, and through PCR was 2.16%. All cases were due to *P. falciparum.* The highest prevalence of infection was found in the Candamo neighborhood. Most infections were asymptomatic and submicroscopic ( Tables 4, 5).

**Table 4.** Estimated prevalence of malaria. Tumaco, July to December 2019.

	Total surveys	Unweighted count	Prevalence (%)	95% Confidence Interval		Standard error (%)
				Lower	Upper	
Microscopy	1,504	11	0.81	0.38	1.74	0.31
Rapid Test	1,504	8	0.62	0.24	1.61	0.29
PCR	1,492	28	2.16	1.58	2.94	0.33
Total	1,504	39	2.97	2.06	4.27	0.54
**Sex**						
Male	531	14	3.21	1.23	8.15	1.51
Female	973	25	2.84	1.94	4.15	0.54
**Age (years)**						
0-9	272	9	4.06	1.38	11.36	2.13
10-19	318	12	4.42	2.37	8.09	1.34
20-49	626	12	2.06	0.55	1.20	3.51
≥50	288	6	2.26	1.00	5.03	0.90
**Symptoms**						
**Microscopy/RDT**						
With symptoms	1,504	6	0.51	0.17	1.51	0.27
Without symptoms	1,504	5	0.31	0.13	0.73	0.13
Total	1,504	11	0.81	0.38	1.74	0.31
**PCR**						
With symptoms	1,492	1	0.05	0.01	0.38	0.05
Without symptoms	1,492	27	2.11	1.53	2.89	0.33
Total	1,492	28	2.16	1.58	2.94	0.33
**Microscopy/RDT/PCR**						
With symptoms	1,504	7	0.56	0.20	1.53	0.28
Without symptoms	1,504	32	2.41	1.77	3.28	0.37
Total	1,504	39	2.97	2.06	4.27	0.54
**Neighborhood**						
Once de Noviembre	159	7	4.40	1.27	14.14	1.93
Obrero	147	2	1.36	0.25	6.94	0.82
Ciudadela	336	9	2.68	0.93	7.48	1.19
Unión Victoria	145	5	3.45	2.08	5.68	0.63
Nuevo Milenio	487	6	1.23	0.50	3.03	0.52
Los Ángeles	159	3	1.89	0.43	7.89	1.00
Candamo	71	7	9.86	0.05	96.20	3.81

**Table 5.** Test results according to symptoms. Unweighted count. Commune 5 neighborhoods, Tumaco (Nariño, Colombia). October 2019.

Test	Symptoms		Total
	Yes	No	
Optical microscopy (RDT)	6 (5)	5 (3)	11 (8)
PCR	1	27	28
Total	7	33	39

In parentheses, the cases diagnosed with RDT.

The symptoms expressed by individuals who tested positive for malaria at the time of the survey were: fever (26.7%, 95% CI 9.9% - 54.7%), shivers (26.7%, 95% CI 9.9% - 54.7%), headache (24.0%, 95% CI 8.1% - 52.9%), diaphoresis (19.2%, 95% CI 5.0% - 51.8%), limb pain (24.3%, 95% CI 7.9% - 54.5%), and weakness (24.3%, 95% CI 7.9% - 54.5%). The mean parasite count of individuals who tested positive for malaria through optical microscopy was 653.5 parasites/μL (95% CI 101.5 to 1,205.5).

Ten individuals had malaria in the last year. Of them, none had malaria in the last month. They also did not have positive test results for malaria in the last 15 days, and they had not consumed antimalarial drugs.

Quality control performed by the National Institute of Health on the samples analyzed through optical microscopy and RDT indicated a 100% positivity rate concordance and 100% negativity rate concordance.

### PCR

Out of the 1,504 blood samples collected, 1,492 were examined through PCR because some samples had very small amounts of blood. The prevalence of amplification of the *pfhrp2* gene was 2.16% (95% CI: 1.58% - 2.94%), and for the *pfhrp3* gene and the *PvSSU* gene it was 0. In the fragment sequences of the gene coding for *pfhrp2*, no polymorphisms were found when compared to the sequences reported in GenBank. We do not have many photographs of uncut gels available. The positive samples were obtained in different assays; there is not a gel with the amplification of all the positive samples.

### Entomological study

The entomological study was conducted in 25 households in the Obrero, Los Ángeles, Candamo, Unión Victoria, Ciudadela, Nuevo Milenio and Once de Noviembre neighborhoods. The presence of immature and adult *Anopheles spp.* mosquitoes was registered.

**Figure 3.**
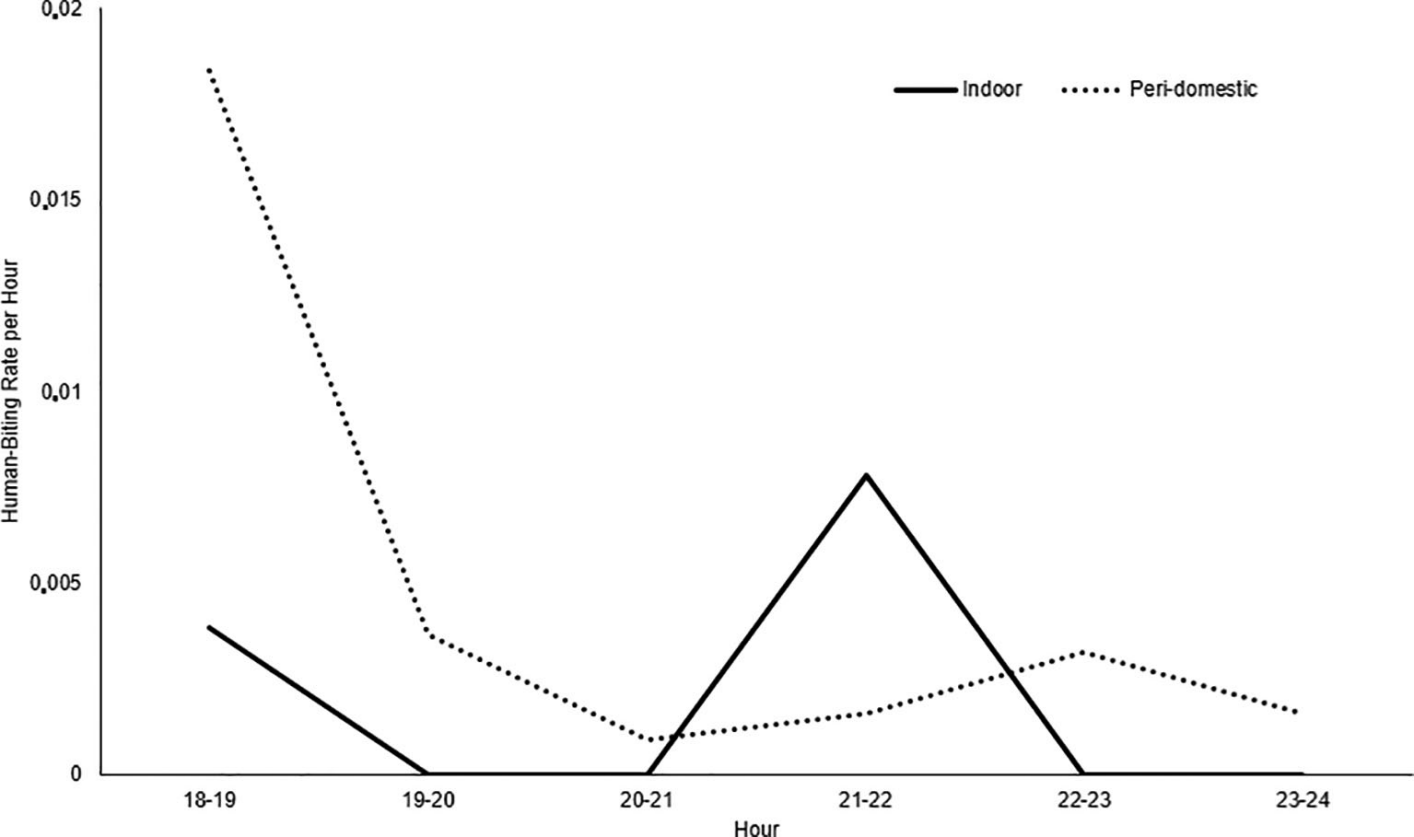
Indoor and Peri-Domestic Human-Biting Rate (HBR). Commune 5 neighborhoods, Tumaco (Nariño, Colombia). October 2019.

37 *Anopheles spp.* mosquitoes belonging to the *albimanus* species were collected. The highest number of mosquitoes was identified in the Los Ángeles and Candamo neighborhoods. No malaria vector mosquitoes were identified in the Nuevo Milenio and Once de Noviembre neighborhoods. Between 18:00 and 19:00 hours, the highest activity occurred in the peri-domestic area, with a 0.018 human-biting rate (HBR) per hour and indoor between 21:00 and 22:00 hours, with a 0.007 HBR ( Figure 3). The average human biting rate (HBR) at night was 0.74 and the HBR was 0.06 for all studied areas.

83 breeding sites were found. In the Commune 5 neighborhoods of Tumaco, *An. albimanus* was found in artificial ponds and wells. Out of those, two had *Anopheles spp.* mosquito larvae, one in the Unión Victoria neighborhood and the other one in the Obrero neighborhood. One breeding site was a pond and the other one was a well. 32 larvae were collected in both breeding sites. 27 of them were in late stages (3rd and 4th) and were identified as *An. albimanus.*

### Related factors

The exploration of associated factors in households where malaria infections occurred is shown in Table 6. The presence of three or more people living in the households (OR 4.98; 95% CI 1.99-12.45) and having three and more sleeping rooms (OR 2.38; 95% CI 1.10-15.13) were suggested as risk factors, and the use of a protective net for sleeping (OR 0.18; 95% CI 0.03-0.97) was suggested as a protective factor. However, the multivariate analysis indicated that households with three or more people had a higher risk of malaria infection (ORa 4.05; 95% CI 1.57-10.43).

**Table 6.** Malaria-related factors in commune 5 households. Tumaco, July to December 2019.

Variable	Prevalence (%)	95% Confidence Interval		Standard error	OR	95% Confidence Interval	
		Lower	Upper			Lower	Upper
Wattle and daub wall							
Yes	0.77	0.20	2.88	0.51	1.47	0.45	4.82
No	6.55	4.64	9.17	1.11			
Brick wall							
Yes	4.49	2.81	7.09	1.03	1.26	0.44	3.60
No	2.83	1.28	6.14	1.10			
Wood wall							
Yes	1.21	0.53	2.74	0.49	0.46	0.18	1.20
No	6.11	3.95	9.33	1.30			
Wall – other materials							
Yes	0.84	0.23	3.07	0.54	1.80	0.42	7.72
No	6.47	4.54	9.16	1.13			
Windows							
Yes	7.14	4.90	10.27	1.31	3.36	0.42	27.17
No	0.18	0.02	1.36	0.18			
Electricity							
Yes	0.00	0.00	0.00	0.00			
No	7.32	5.04	10.50	1.33	-	-	-
Gas connection							
Yes	0.49	0.07	3.44	0.48	2.05	0.23	18.20
No	6.82	4.86	9.49	1.13			
Water							
Yes	2.48	1.22	4.98	0.87	1.39	0.72	2.66
No	4.84	3.38	6.88	0.85			
Sewage							
Yes	7.32	5.04	10.50	1.33	-	-	-
No	0.00	0.00	0.00	0.00			
Garbage collection							
Yes	0.55	0.13	2.33	0.40	0.86	0.21	3.62
No	6.77	4.56	9.94	1.31			
People living in the household							
3 or more	5.87	4.02	8.50	1.09	4.98	1.99	12.45
1 or 2	1.45	0.66	3.17	0.57			
Rooms where people sleep							
3 or more	4.57	2.96	6.98	0.97	2.38	1.10	5.13
1 or 2	2.75	1.50	5.00	0.82			
Use of protective net while sleeping							
Yes	6.74	4.52	9.95	1.32	0.18	0.03	0.97
No	0.58	0.17	1.90	0.34			

The exploration of risk factors for individuals with malaria infection is presented in Table 7. None of the evaluated factors was statistically associated with a higher probability of having malaria.

**Table 7.** Malaria-related factors in commune 5 inhabitants in Tumaco, July to December 2019.

Variable	Prevalence (%)	95% Confidence Interval		Standard error	OR	95% Confidence Interval	
		Lower	Upper			Lower	Upper
**Age (years)**							
0-14	1.1	0.5	2.4	0.4	1.00		
15 or more	1.9	1.4	2.5	0.3	1.50	0.67	3.35
**Sex**							
Male	1.1	0.5	2.9	0.5	1.00		
Female	1.8	1.3	2.7	0.3	1.14	0.35	3.66
**Income**							
1 or more MLW	0.4	0.2	1.0	0.2	1.00		
<1 MLW	2.6	1.6	4.2	0.6	1.71	0.54	5.37
**Education**							
Some	3.0	2.1	4.3	0.5	1.00		
None	0.0	0.0	0.0	-	-	-	-
**Civil status**							
Has a partner	0.8	0.5	1.3	0.2	1.00		
No partner	2.1	1.3	3.4	0.5	0.59	0.32	1.09
**Time living in the household**							
10 or more years	1.6	1.1	2.3	0.3	1.00		
< 10 years	1.4	0.7	2.8	0.5	1.22	0.57	2.60
**Malaria episodes – last year**							
Yes	0.0	0.0	0.0	-	-	-	-
No	3.0	2.1	4.3	0.5	-	-	-
**Pregnant**							
Yes	0.1	0.0	0.6	0.1	1.00		
No	2.9	2.0	4.2	0.5	2.54	0.27	24.11
**Have you taken antimalarial drugs over the last 15 days?**							
Yes	0.1	0.0	0.6	0.1	1.00		
No	2.9	2.0	4.2	0.5	12.81	0.93	176.45
**Ethnic group**							
African descent	3.0	2.1	4.3	0.5	1.00		
Others	0.0	0.0	0.0	-	0.0	-	-

## Discussion

This study conducted in Commune 5, urban area of Tumaco, estimated malaria prevalence, *pfhrp2* gene amplification, and explored factors associated with infection. The prevalence of malaria was 2.97%. It was higher in the 10 to 19 age group (4.42%) and 0 to 9 (4.06%), decreased with age, and was higher in cases of asymptomatic and submicroscopic infection. The *pfhrp2* gene was found and the disease was positively associated with households inhabited by three or more people.

The blood samples were taken during the dry season, between July and September 2019. Malaria in the urban area of Tumaco has not shown a clear seasonal behavior.

The study found malaria in Commune 5, an area believed to have high transmission according to reports from the Vector-Borne Disease Prevention and Control Program in the Nariño Department (API 2014 84.0 cases per 1,000 inhabitants; API 2015 220.5 and API 2016 165.8) (Pilar Perez, Vector-Borne Disease Prevention and Control Program in the Nariño Department, personal communication). The decrease in the prevalence of malaria over time is possibly due to the intensification of actions to search for and eliminate breeding sites, massive use of mosquito nets, and social participation as measures implemented for Commune 5. Diagnosis and treatment actions also have contributed, despite the difficulties that exist in accessing services. In this way, it has been reduced human contact (infected) —a vector that has contributed to the decline in the burden of this disease.

The higher prevalence of malaria in people under 20 years of age suggests that the infection may have been acquired locally. Unlike Africa, which has a high transmission and where the population, most affected in terms of morbidity and mortality is under 5 years of age, in areas of low transmission such as Colombia, malaria occurs in older age groups. Among the aspects that are considered to think that the transmission occurs in the urban area are confirming that the infection in the residents of these areas; evidence of the presence of adult *Anopheles spp.* that are biting the inhabitants of urban dwellings and identifying breeding sites near them. ^40^

In South America, the prevalence of symptomatic and asymptomatic infections have ranged from 0.1% to 33% in urban areas of Porto Velho, ^16^ Manaos, ^41^ and Mâncio Lima ^42^ in Brazil. In Colombia, prevalence has ranged from 0% to 5.8%. In Quibdó, the estimated asymptomatic prevalence in a group of schoolchildren was 0% (95% CI: 0-1.4). ^43^ Two cases of submicroscopic infection were reported in Yesquita, Silencio and Roma neighborhoods of Quibdó. ^44^ In the Santa Mónica de Guapi neighborhood (Cauca Department, on the Colombian Pacific), four successive measurements reported asymptomatic prevalence of 2.7%, 1.2%, 0.6% and 0.3%, respectively. ^27^ In Buenaventura, the prevalence was 4.4% ^21^ and in the California neighborhood in Tumaco it was 5.8%. ^26^ These figures are not comparable with those reported in this study due to differences in terms of study design, type of sampling used, selection of individuals, diagnostic techniques, time of year in which the study was conducted, and the area where it was carried out. Concerning this last aspect, although there is no consensus on the definition of urban and peri-urban, the presence of symptomatic and asymptomatic malaria in Commune 5 of Tumaco was demonstrated, turning it into a potential human reservoir and a challenge for disease elimination strategies.

The persistence of urban malaria may be caused by people moving back and forth between urban and rural areas. People travel from urban to rural areas, where the endemic is higher, either for work or leisure. People also travel from endemic rural areas to urban areas to access health services or to engage in commercial activities, causing them to stay for a long period of time there. ^15^ Rural areas are the main source of malaria infection in urban areas. ^45^

In urban areas, malaria infection is not expected to be prevalent due to better-designed households with better basic utilities, better access to health services, and the absence of mosquito breeding sites. ^34^^,^^46^ However, in the study area, most households were found to belong to a low socioeconomic stratum, lack sewage, have few mosquitoes and breeding sites, and have low biting rates, which could in part contribute to disease transmission.

In this study, households with higher occupancy showed higher malaria risk. Studies in Rwanda and Tanzania found that mosquitoes are more attracted to houses with many people ^47^^,^^48^ because there is a higher production of cues that attract them and increase the risk of transmission, compared to households with lower occupancy. ^49^

Something that stands out is the high percentage of households in which people slept with nets (98%). Although no distinction was made between untreated nets, those treated at some point in time, and those treated with insecticide, the bivariate analysis allows us to see their protective effect when households with and without malaria cases were compared. If nets are a major part of malaria elimination strategies, implementation, monitoring and evaluation of their use and efficacy should be routinely carried out in Commune 5 of Tumaco. ^50^

In terms of case detection, as in other studies, PCR had the highest diagnostic performance, ^40^ followed by microscopy. When comparing microscopy/RDT results with PCR results, lack of concordance was observed. Those with a positive diagnosis through microscopy/RDT were not detected through PCR and those with a positive diagnosis through PCR were not detected through microscopy/RDT. It is possible that people who were positive according to microscopy/RDT did not have the parasite's DNA so it could not be detected through PCR, as described by Mudare *et al.,* ^51^ or the sample may have had a low amount of parasitic DNA or it may have degraded. Another possibility is that there were changes in the primer ringing site, which would prevent amplification of the gene fragment under study. On the other hand, a positive microscopy with a negative RDT is probably because the limit of detection of RDT. The same rational is true for negative RDT with positive PCR. Cases of negative thick smear with positive RDT could be explained because *Pfhrp2* antigen could be detected in the blood until 30 days after a good malaria treatment.

Specifically, for negative PCR results it can be inferred that:

1. The ringing site of the primers may have some modification that does not allow their hybridization.
2. Although the degradation of genomic DNA is improbable because the verification of its quality and integrity was carried out through quantification and the 260/280 ratio, which for all DNA was between 1.8 and 2.0 and by electrophoresis, respectively, but it is not unlikely that the reactions have contaminants that interfere with amplification.
3. The sequences of the selected fragments may contain variable regions that are not recognized by the designed primers.

The species detected through the various diagnostic tests was *P. falciparum*, a finding that also corresponds to the most frequently reported species for malaria on the Colombian Pacific coast. ^4^

In this study, differences were found in terms of the prevalence of malaria among men and women. As reported in other studies, malaria was more prevalent in men. This could be due to the fact that they spend more time doing outdoor activities, arrive to their households late in the evening, and have an indifferent attitude towards malaria prevention. ^52^

In terms of age groups, the highest prevalence of malaria was observed in the 10 to 19 age group and as age increased, prevalence decreased. This result is similar to that reported in other studies. Older people are likely to have a lower risk of becoming ill because they may develop some immunity if they have been previously exposed to the infection. ^20^^,^^21^ Less than 1% of participants had malaria in the last year and none in the last month. It is possible that in areas of low endemicity, such as Colombia, the lowest prevalence in older age groups is due to the fact that the population that may have less exposure was interviewed. For example, women who remain at home dedicated to household chores and individuals of school age who go to school. Recent studies in areas with high and low malaria transmission rates have revealed that asymptomatic infections contribute to malaria transmission. ^53^ Furthermore, asymptomatic malaria may precede symptomatic disease, and may confer partial immunity against infection. ^54^ In this study, participants were sampled at a single point in time. Those who were classified as asymptomatic had no history of illness in the last month and had not used antimalarial drugs in the last 15 days. For them, it is difficult to establish if they were in the incubation period of the disease or if they were carriers of a low number of parasites. Resolving a situation like this requires follow-up. In this regard, a study of 268 people followed for 29 months in western Kenya, in a high-transmission area, discovered that asymptomatic infections were highly likely to be followed by symptomatic disease. ^55^ However, this does not seem to be the case in Commune 5 of Tumaco, which has a low transmission and where complicated malaria and malaria mortality do not occur.

This study found the presence of the *pfhrp2* gene in samples from the urban area of Commune 5 in Tumaco. The findings suggest that a low proportion of malaria infections are not detected through the RDTs used in Colombia. In South America, gene deletions have been reported in Peru (prevalence between 20.7% and 41.0%) and Brazil (one case). ^56^ In Colombia, these deletions have been reported in the Amazonas Department, where they reached a prevalence of 67% ^57^; in Guapi (Cauca Department), where 6% of the parasite simples analyzed showed *pfhpr2/3* gene deletion. ^27^ The factors promoting the appearance of these parasites in some regions still remain unknown. ^58^ The estimates obtained in this study do not exceed the minimum WHO criteria (>5%); however, it is important to monitor the hrp2-based RDTs used in the country. On the other hand, continuous monitoring related to submicroscopic infections and negative pfhrp2 results should be performed when using RDT. It is suggested that submicroscopic infections with parasites with *pfhrp2/3* gene deletion could have infectious potential for mosquitoes and thus favor malaria transmission. ^57^

In the context of our study due to insufficient resources, we did not evaluate the deletion of these genes, we only amplified a fragment of these genes by means of PCR. However, in order to improve the management of clinical cases and design strategies for diagnosis, the detection of deletions in the *hrp2/3* genes is relevant, since, as has been described, these can affect the accuracy of PDR in malaria endemic regions and of course in malaria control programs.

On the other hand, in the Commune 5 neighborhoods of Tumaco, *An. albimanus* was found in artificial ponds and wells, as described in the Buenaventura urban area. ^20^ Breeding sites were found in all the neighborhoods that were part of the study, except in Nuevo Milenio and Once de Noviembre. The differences between neighborhoods are caused by the urbanization level. Stilt houses predominate in some neighborhoods, while there is no sewage system in others, which leads to the construction of ditches where water stagnates and mosquitos breed. Although there is ongoing entomological surveillance, mosquito control interventions need to be strengthened and the community should be involved. *An. albimanus* had greater hematophagic activity in peri-domestic areas between 18:00 and 19:00 hours, a time when people engage in social activities outdoors and when they can possibly contract the disease, ^21^ and indoors, between 21:00 and 22:00. However, given the low densities of *An. albimanus* and the limited observation, it was not possible to determine whether the mosquito was highly active at other times at night.

*An. albimanus* is considered a primary malaria vector in Colombia and particularly in Tumaco. ^59^ The presence of *An. albimanus* in different neighborhoods of Commune 5 provides partial evidence of the possible local transmission of malaria. This mosquito has been considered an opportunistic species due to its ability to adapt to different breeding sites. ^60^ Despite the small number of adults collected, there is a risk of transmission, taking into account that the maximum biting activity occurs in the peridomicile between 18:00 and 19:00 hours.

This study results should be studied with caution, given the limitations. First, the samples to be analyzed through PCR and *pfhrp2/3* gene detection were taken on filter paper and were extracted after prolonged storage (due to the prioritization of PCR for Covid-19), which may have limited the amount of DNA to be used or may have promoted its degradation. Second, some samples collected on filter paper had little blood so the amount of gDNA was also variable. Third, the vast majority of individuals detected who were found to have malaria had no symptoms, which may not have allowed the researchers to detect the prevalence of *pfhrp2/3* genes in symptomatic individuals. Fourth, the presence of illegal armed groups did not allow for an adequate entomological study because only partial observation was allowed in some neighborhoods, while in others the research group was not allowed. Fifth, due to study design, the participants were only questioned at sampling and there was no follow-up, so it is not possible to know if those who were asymptomatic later became symptomatic. Sixth, during survey application a memory bias may have been present, which could have altered the respondents' way of answering the questions.

Particular attention requires the limitation related to entomological aspects. The results presented are only an approximation to the subject. The difficulties presented did not allow them an adequate study of the ecology of the vector and to characterize the aspects related to the vector's biting density, feeding patterns, biting activity, parity rates, and resting behavior, among other issues that represent important factors for malaria transmission within the Commune 5.

A strength of this research was the participation of the “Vector-Borne Disease Prevention and Control Program in the Nariño Department” officials and the municipality of Tumaco. Their intervention will promote the use of these results for decision making in activities related to the pre-elimination of urban malaria in Tumaco.

In summary, the prevalence of malaria was estimated at 2.97% and the prevalence of asymptomatic infection was 2.16% in the urban area of Commune 5 of Tumaco. Parasites positive for the *pfhrp2* gene were found. *An. albimanus* larvae were found in breeding sites and adult forms were found indoors and in peri-domestic areas. All of the cases were caused by *P. falciparum.* The disease was positively associated with households inhabited by three and more people. Based on these results, pre-elimination of urban malaria should include early diagnosis strategies and continuous active surveillance of asymptomatic infection, which requires the inclusion of molecular diagnostic tests. In addition, the RDTs used to diagnose should be monitored for potential *pfhrp2* gene deletion, which will be focused on considering alternatives. Additionally, work should be coordinated with other sectors to establish activities aimed at improving basic sanitation and conduct continuous educational activities aimed at preventing the disease.

## Data availability

### Underlying data

Zenodo: Malaria Prevalence in Commune 5 in Tumaco (Nariño, Colombia). https://doi.org/10.5281/zenodo.6395340 ^61^

This project contains the following underlying data:

- Malaria Prevalence in Commune 5 in Tumaco – house – readme.xlsx
- Malaria Prevalence in Commune 5 in Tumaco – house.xlsx
- Malaria Prevalence in Commune 5 in Tumaco – persons – readme.xlsx
- Malaria Prevalence in Commune 5 in Tumaco – persons.xlsx
- Breeding sites.xlsx
- Determination Anopheles.xlsx
- Malaria gel images.pdf

### Extended data

Zenodo: Malaria Prevalence in Commune 5 in Tumaco (Nariño, Colombia). https://doi.org/10.5281/zenodo.6395340 ^61^

This project contains the following extended data:

- Housing survey.docx
- Housing survey English.docx
- People survey.docx
- People survey English.docx

Data are available under the terms of the Creative Commons Attribution 4.0 International license (CC-BY 4.0).
