# Supplementary material for: Malaria prevalence in Commune 5 in Tumaco (Nariño, Colombia)
Source: F1000Res. 2023 Jul 19;11:448. Originally published 2022 Apr 21. [Version 3] doi: 10.12688/f1000research.110361.3 (PMC10913070; doi:10.12688/f1000research.110361.3)
Supplement: Peer review history

# Reviewer report: Reviewer response for version 3

**DOI:** 10.5256/f1000research.144551.r245104

**Published:** 2024-03-01

**Authors:** Ebenezer Krampah Aidoo

**Affiliations:** 1 Accra Technical University, Accra, Ghana

The authors conducted a study in Tumaco, Columbia “aimed at estimating malaria prevalence, detecting pfhrp 2/3 genes, and exploring associated factors in the urban area (Commune 5) in Tumaco during 2019”. Generally, the manuscript is well written. However, the authors can further improve the manuscript by considering these suggestions.

**Title:   “**Malaria prevalence in Commune 5 in Tumaco (Nariño, Colombia)”. Consider **a broader title that captures the essence of the aim of the study.**

**Abstract (**Abstract in its original version was translated into English by the reviewer to get a quick overview of the study)

**Background**

 “pfhrp 2/3” should be written in full for first-time usage. Thereafter, it can be shortened.

**Methods**

 Rapid diagnostic testing (RDT) should read **rapid diagnostic test (RDT) kits.**

**Results**

 Consider starting with **words (**One thousand five hundred and four individuals) **other than figure** as in 1,504 people.  2.97% (95% CI: 2.1 - 4.3%) should be without the percent at the end of the bracket as in **2.97% (95% CI: 2.1 - 4.3). **The same should be for 2.16% (95% CI: 1.6 - 2.9%). “(adjusted odds ratio [aOR]”, should be adjusted odds ratio [ **AOR**]. For consistency, maintain one of these formats below throughout the manuscript.

**(95% CI: 2.1 - 4.3) or 95% confidence interval [CI] 1.57-10.43)**

 P. falciparum. ***Plasmodium*** should be written in full for first-time usage. Thereafter, it can be shortened and italicized.

**Introduction**

 “……. aimed at estimating malaria prevalence, detecting pfhrp 2/3 genes, and exploring associated factors in the urban area (Commune 5) in Tumaco during 2019”. Please clarify what you mean by **associated factors. Specificity is key.**

**Ethics and consent**

 “This study was approved by the ethics committees of the National Institute of Health (April 4, 2017, Minutes) and Fundación Santa Fe de Bogotá (April 22, 2019, Minutes)”. Consider quoting the reference number issued by the committee and as used in your ethical approval letter.

**Study area.**

 “The presence of *Anophelines spp*.” should read The Presence of *Anopheles* spp.

**Laboratory procedures**

**Optical microscopy**

 “Blood samples were **taken through** finger puncture with a sterile lancet and each sample was then put on a clean film”.  Consider rewording the sentence. Blood samples were **collected** **by** and not **taken through**. What do you mean by each “sample was then put on a clean film”? Use a trained malaria microscopist rather than a Bacteriologist.

**Polymerase chain reaction (PCR)**

**“** **10 mm 2** of Whatman #3 filter paper……”. Make the necessary correction.

**Standardization of protocols for genomic DNA extraction from filter paper samples**

 Could you provide references for the saponin/chelex-100 method of DNA extraction and conventional PCR used?

**Definitions**

 Move the definition of the 3 terminologies on page 7 to the introduction.

 Page 11 “ **In the last year**, 0.67% of the participants reported they had had malaria”. State the year wherever it appears in the manuscript.

**Prevalence of malaria**

 “The prevalence obtained through microscopy/RDT was 0.81%”. From Table 4, it’s true for microscopy but not so for RDT. How do you explain this?

 How do you reconcile these two statements below?

 “The prevalence was **established** from 1,504 microscopic examinations, 1,504 rapid diagnostic tests, and **1,504 PCR**”. 

 “Out of the 1,504 blood samples collected, **1,492 were examined through PCR** because some samples had very small amounts of blood”.

**Martha Cecilia Suárez-Mutis** **(one of the reviewers)** asked “In total, how many samples were actually positive? What is the denominator? Table 5 shows 39 positive samples. If the denominator is 1,504, the prevalence was 2.59 and not 2.97%. Please add all the information and clarify the number and prevalence of positive samples’’. Find below your response:

 “The prevalence of 2.97% corresponds to the stratified multistage random sampling with proportional assignment used by us. We have indicated this in the methods section. The prevalence of 2.59% would correspond to a simple random sampling that was not used in this study”.

 How you arrived at the above analyses should be **“captured” **in the statistical analysis section of the manuscript and also indicated as a **footnote as part of Table 4**

**Discussion**

 Page 16 “Concerning this last aspect, although there is no consensus on the definition of urban and peri-urban” .............Reference will be needed.

 Page 17 - “Less than 1% of participants had malaria in the last year and none in the last month”. Be specific about the year and month wherever it appears.

 Page 17 “………where 6% of the parasite **simples **analyzed showed pfhpr2/3 gene deletion”. Should be samples.

 Page 17 PDR, Is it PCR? If not, write in full.

 Page 17 “This study results should be studied with caution, given the limitations”. Consider using limitations of the study include…….

 Page 18- “Particular attention requires the limitation related to entomological aspects. The results presented are only an approximation to the subject”. The statement should be reworded so the meaning comes out clearer.

 “In addition, the RDTs used to diagnose should be monitored for potential pfhrp2 gene deletion, which will be focused on considering alternatives”. What do you mean? Please explain further.

**General observation**

 Were you able to detect pfhrp 3 genes and explore the associated factors?

 “Plasmodium falciparum specific RDTs specifically recognize HRP2 antigen that encodes for the pfhrp2 gene and whose antibodies cross-react with histidine-rich protein 3 (pfhrp3) antibodies due to high degree of similarity in amino acid sequence” (WHO. Malaria Policy Advisory Committee Meeting, 14–16 September 2016, Background document for Session 7; *P. falciparum* hrp2/3 gene deletions, conclusions and recommendations of a technical consultation. Geneva: World Health Organization, 2016. Malaria Policy Advisory Committee meeting report (September 2016) (who.int))

 The associated factors should be delineated from **Tables 6 and 7** to inform the discussion and **not generalized**.

 The quality of the language will have to be improved.

Is the work clearly and accurately presented and does it cite the current literature?

Yes

If applicable, is the statistical analysis and its interpretation appropriate?

I cannot comment. A qualified statistician is required.

Are all the source data underlying the results available to ensure full reproducibility?

Yes

Is the study design appropriate and is the work technically sound?

Yes

Are the conclusions drawn adequately supported by the results?

Partly

Are sufficient details of methods and analysis provided to allow replication by others?

Yes

Reviewer Expertise:

My research interests are in the areas of malaria epidemiology, host-parasite interactions, dynamics of disease transmission, antimalarial drug resistance and use of molecular tools to better understand the transmission of malaria, other infectious diseases (COVID-19, Helicobacter pylori infection etc) and neglected tropical diseases (Schistosomiasis, Soil-transmitted helminth infection etc).

I confirm that I have read this submission and believe that I have an appropriate level of expertise to confirm that it is of an acceptable scientific standard, however I have significant reservations, as outlined above.

# Reviewer report: Reviewer response for version 2

**DOI:** 10.5256/f1000research.138123.r149865

**Published:** 2022-10-12

**Authors:** Martha Cecilia Suárez-Mutis

**Affiliations:** 1 Instituto Oswaldo Cruz, Laboratório de Doenças Parasitárias, Fundação Oswaldo Cruz, Rio de Janeiro, Brazil

Some questions were not answered by the authors and thus, new doubts arose: the main question is in the interpretation of pfhrp2/3 prevalences (and malaria prevalence):

 1) In total, how many samples were actually positive? What is the denominator? table 5 shows 39 positive samples. If denominator is 1,504, the prevalence was 2.59 and not 2.97%. Please add all the information and clarify the number and prevalence of positive samples.

 On the other hand, it is assumed that all *P. falciparum* should have the pfhrp2/3 gene. Its absence indicates a deletion of this gene or that the PCR used was not able to detect it. What was the PCR protocol used for detection of *pfhrp*2/3?. Please, add the reference. These data are extremamely important for quality of the work. Was it and "i *n house*" method?. If this was the case, the complete protocol must be added to methods sections. 

 It is probably that authors used as denominator for *pfhrp*2 gene detection, almost all the samples collected (n=1.492). As authors only found 39 *P. falciparum* positive samples, the denominator for *pfhrp* amplification should be 39 (as in table 5) and not 1.492. Maybe this is the cause of finding only 2.16% *pfhrp *gene amplification. As data are incomplete this is an assumption. Authors must clarify. It is very confusing. 

 Minor English and taxonomic corrections are necessary:

 All taxonomic names with only generic names must have spp. at the end (ex. *Anopheles* spp.).

 page 6/40: 

 "In this city, 70%of malaria cases are reported from Commune 5 in the last ten years".

 As authors are talking about malaria in the last ten years (in past), the verb should be written in past time: ...In this city, 70%of malaria cases **were** reported..;

 Page 6/40: 

 " *Polymerase chain reaction (PCR)*

 For PCR, two drops of blood were obtained from the same finger puncture and were then put on the filter paper. 10 mm ^2^ of Whatman #3 filter paper impregnated with blood were taken and genomic DNA extraction was performed according to the protocol described in the article".

 Authors did not give information about this article. Please add a reference.

 Pag 6/40:

 Authors said that: "Standardization of protocols for genomic DNA extraction from whole blood and filter paper samples". However, methods only explain about DNA extraction and PCR from filter paper samples. No mention about whole blood experiments was found in the manuscript. If authors collected whole blood samples (not filter paper) these data should be in the methods. If not, this part of the sentence should be excluded. 

 Page 7/40: 

 The sentence: "For the amplification of the different fragments of the genes mentioned, we designed the specific primers and the PCR conditions for each of them were standardized. For the design of the primers, the GC content, dimer formation, loop formation, palindromes and Tm were taken into account. The sequences of the HRP2/HRP3 and PvSSU genes published in the Genbank were used, an alignment was performed using clustalW (Refseq) and the primers were designed from conserved regions of the sequences obtained" is repeated at the end of page 6. Please excluded the repeated sentence.

 Discussion:

 Page 16/40: Authors said: "In terms of case detection, as in other studies, PCR had the highest diagnostic performance, ^40^ followed by microscopy. When comparing microscopy/RDT results with PCR results, lack of concordance was observed. Those with a positive diagnosis through microscopy/RDT were not detected through PCR and those with a positive diagnosis through PCR were not detected through microscopy/RDT. It is possible that people who were positive according to microscopy/RDT did not have the parasite's DNA so it could not be detected through PCR, as described by Mudare *et al.,* ^51^ or the sample may have had a low amount of parasitic DNA or it may have degraded. Another possibility is that there were changes in the primer ringing site, which would prevent amplification of the gene fragment under study.". This part is a little confusing. For better clarification, we suggested to separate RDT results, thick smear results and PCR results and to discuss each finding (negative RDT with positive PCR, etc.).

Is the work clearly and accurately presented and does it cite the current literature?

Yes

If applicable, is the statistical analysis and its interpretation appropriate?

Partly

Are all the source data underlying the results available to ensure full reproducibility?

Yes

Is the study design appropriate and is the work technically sound?

Partly

Are the conclusions drawn adequately supported by the results?

Partly

Are sufficient details of methods and analysis provided to allow replication by others?

Yes

Reviewer Expertise:

Malaria epidemiology and molecular epidemiology of Plasmodium infections; malaria diagnosis, Pfhrp2/3 deletion detection; asymptomatic Plasmodium infections.

I confirm that I have read this submission and believe that I have an appropriate level of expertise to confirm that it is of an acceptable scientific standard, however I have significant reservations, as outlined above.

# Response

**Published:** 2023-07-17

**Authors:** Pablo Enrique Chaparro Narváez

**Affiliations:** Instituto Nacional de Salud (Colombia), Colombia

Thank you for your new review. These are our answers:

 Some questions were not answered by the authors and thus, new doubts arose: the main question is in the interpretation of pfhrp2/3 prevalence’s (and malaria prevalence):

- 1) In total, how many samples were actually positive? What is the denominator? table 5 shows 39 positive samples. If denominator is 1,504, the prevalence was 2.59 and not 2.97%. Please add all the information and clarify the number and prevalence of positive samples.

**Answer:** The prevalence of 2.97% corresponds to the stratified multistage random sampling with proportional assignment used by us. We have indicated in the methods section. The prevalence of 2.59% would correspond to a simple random sampling that was not used in this study.

- On the other hand, it is assumed that all *P. falciparum* should have the pfhrp2/3 gene. Its absence indicates a deletion of this gene or that the PCR used was not able to detect it. What was the PCR protocol used for detection of pfhrp2/3?. Please, add the reference. These data are extremely important for quality of the work. Was it and "in house" method?. If this was the case, the complete protocol must be added to methods sections.

**Answer:** As indicated in the methods section, PCRs were performed for the individual amplification of the HRP2 and HRP3 genes. It is not possible to confirm the deletion of these genes with this study, since the sequencing of the complete genes was not carried out, but only fragments of them.

- It is probably that authors used as denominator for pfhrp2 gene detection, almost all the samples collected (n=1.492). As authors only found 39 *P. falciparum* positive samples, the denominator for PfHRP amplification should be 39 (as in table 5) and not 1.492. Maybe this is the cause of finding only 2.16% PfHRP gene amplification. As data are incomplete this is an assumption. Authors must clarify. It is very confusing.

**Answer:** The prevalence for the detection of the pfhrp2 gene was also calculated taking into account multistage stratified random sampling with proportional assignment.

- Minor English and taxonomic corrections are necessary:
- All taxonomic names with only generic names must have spp. at the end (ex. Anopheles spp.).

**Answer**: The adjustment was made on pages 9, 15, 16, 19.

- page 6/40:

"In this city, 70% of malaria cases are reported from Commune 5 in the last ten years".

 As authors are talking about malaria in the last ten years (in past), the verb should be written in past time: ...In this city, 70% of malaria cases were reported.

**Answer:** The adjustment was made.

- Page 6/40:

"Polymerase chain reaction (PCR)

 For PCR, two drops of blood were obtained from the same finger puncture and were then put on the filter paper. 10 mm2 of Whatman #3 filter paper impregnated with blood were taken and genomic DNA extraction was performed according to the protocol described in the article".

 Authors did not give information about this article. Please add a reference.

**Answer:** The adjustment was made.

- Pag 6/40:

Authors said that: "Standardization of protocols for genomic DNA extraction from whole blood and filter paper samples". However, methods only explain about DNA extraction and PCR from filter paper samples. No mention about whole blood experiments was found in the manuscript. If authors collected whole blood samples (not filter paper) these data should be in the methods. If not, this part of the sentence should be excluded.

**Answer**: The adjustment was made. The whole blood extraction was eliminated since it is not mentioned in the manuscript.

- Page 7/40:

The sentence: "For the amplification of the different fragments of the genes mentioned, we designed the specific primers and the PCR conditions for each of them were standardized. For the design of the primers, the GC content, dimer formation, loop formation, palindromes and Tm were taken into account. The sequences of the HRP2/HRP3 and PvSSU genes published in the Genbank were used, an alignment was performed using clustalW (Refseq) and the primers were designed from conserved regions of the sequences obtained" is repeated at the end of page 6. Please excluded the repeated sentence.

**Answer:** Removed the repeated paragraph.

- Discussion:

Page 16/40: Authors said: "In terms of case detection, as in other studies, PCR had the highest diagnostic performance,40 followed by microscopy. When comparing microscopy/RDT results with PCR results, lack of concordance was observed. Those with a positive diagnosis through microscopy/RDT were not detected through PCR and those with a positive diagnosis through PCR were not detected through microscopy/RDT. It is possible that people who were positive according to microscopy/RDT did not have the parasite's DNA so it could not be detected through PCR, as described by Mudare et al.,51 or the sample may have had a low amount of parasitic DNA or it may have degraded. Another possibility is that there were changes in the primer ringing site, which would prevent amplification of the gene fragment under study.". This part is a little confusing. For better clarification, we suggested to separate RDT results, thick smear results and PCR results and to discuss each finding (negative RDT with positive PCR, etc.).

**Answer:** The adjustment was made.

 The correlation between techniques is made to be able to explain why there is not a 100% positivity between tests.

 Specifically, for negative PCR results it can be inferred that:

 1. The ringing site of the primers may have some modification that does not allow their hybridization.

 2. Although the degradation of genomic DNA is improbable because the verification of its quality and integrity was carried out through quantification and the 260/280 ratio, which for all DNA was between 1.8 and 2.0 and by electrophoresis, respectively, but it is not unlikely that the reactions have contaminants that interfere with amplification.

 3. The sequences of the selected fragments may contain variable regions that are not recognized by the designed primers.

# Reviewer report: Reviewer response for version 2

**DOI:** 10.5256/f1000research.138123.r149866

**Published:** 2022-09-09

**Authors:** Meor Termizi Farah Haziqah

**Affiliations:** 1 School of Biological Sciences, Universiti Sains Malaysia, Penang, Malaysia

No further comments are needed. Thank you.

Is the work clearly and accurately presented and does it cite the current literature?

Yes

If applicable, is the statistical analysis and its interpretation appropriate?

Yes

Are all the source data underlying the results available to ensure full reproducibility?

Yes

Is the study design appropriate and is the work technically sound?

Yes

Are the conclusions drawn adequately supported by the results?

Yes

Are sufficient details of methods and analysis provided to allow replication by others?

Yes

Reviewer Expertise:

Parasitology

I confirm that I have read this submission and believe that I have an appropriate level of expertise to confirm that it is of an acceptable scientific standard.

# Reviewer report: Reviewer response for version 1

**DOI:** 10.5256/f1000research.121958.r135483

**Published:** 2022-05-23

**Authors:** Meor Termizi Farah Haziqah

**Affiliations:** 1 School of Biological Sciences, Universiti Sains Malaysia, Penang, Malaysia

This paper discusses malaria prevalence based on several methods (microscopy examination, RDT, and PCR), detection of *pfhrp* 2/3 genes as well as explores the associated factors for malaria infection in the urban area (Commune 5) in Tumaco, Colombia in 2019. The main contribution of the paper is mainly on the information on malaria infection in Tumaco, Colombia with the most recent status of malaria infection was found to be very low with only 2.97% prevalence out of 1,504 individuals being examined. Other than that, it was found that all the positive cases were caused by *Plasmodium falciparum* and this study also manages to detect the main vector species for this parasite which is the *Anopheles albimanus*. The larvae were detected in a pond and a well whereas the adults were detected indoors and in peri-domestic areas.

 However, there are a few suggestions that need to be considered such as follows:

1. Figure 1: it is advisable to have a better mapping as the map provided is not clearly demonstrating the study areas. For example, putting the legends on the map indicating Commune 5 and the map becomes more legible.
2. The total number of samples for each of the laboratory procedures (i.e. techniques used to detect malaria) need to be stated clearly.
3. It would be better and more understandable to include a schematic diagram of the study sampling technique.
4. As for DEFINITION in methods, reword the sentence and provide it in the form of a paragraph which explains in detail the definition used in this paper.

Therefore, I recommend that this paper be Approved after minor revision.

Is the work clearly and accurately presented and does it cite the current literature?

Yes

If applicable, is the statistical analysis and its interpretation appropriate?

Yes

Are all the source data underlying the results available to ensure full reproducibility?

Yes

Is the study design appropriate and is the work technically sound?

Yes

Are the conclusions drawn adequately supported by the results?

Yes

Are sufficient details of methods and analysis provided to allow replication by others?

Yes

Reviewer Expertise:

Parasitology

I confirm that I have read this submission and believe that I have an appropriate level of expertise to confirm that it is of an acceptable scientific standard.

# Response

**Published:** 2022-08-31

**Authors:** Pablo Enrique Chaparro Narváez

**Affiliations:** Instituto Nacional de Salud (Colombia), Colombia

Thank you for your review. These are our answers for your review:

 Figure 1: it is advisable to have a better mapping as the map provided is not clearly demonstrating the study areas. For example, putting the legends on the map indicating Commune 5 and the map becomes more legible.

 A: We have shown the study area on the map and have included the legends

 The total number of samples for each of the laboratory procedures (i.e. techniques used to detect malaria) need to be stated clearly.

 A: The prevalence was established from 1,504 microscopic examinations, 1,504 rapid diagnostic tests, and 1,504 PCR.

 It would be better and more understandable to include a schematic diagram of the study sampling technique.

 A: We have included the schematic diagram of the study's sampling technique

 As for DEFINITION in methods, reword the sentence and provide it in the form of a paragraph which explains in detail the definition used in this paper.

 A: This study assumed the definitions of symptomatic malaria, asymptomatic malaria, and submicroscopic malaria. Symptomatic malaria corresponded to an individual with positive microscopy/RDT and symptoms of malaria, such as fever, chills, vomiting, convulsions, malaise, headache and/or loss of appetite. Asymptomatic malaria corresponded to an individual with positive microscopy/RDT or PCR, without symptoms. ^38,39^. Submicroscopic malaria corresponded to an individual with positive PCR and negative microscopy/RDT. Submicroscopic infections are almost exclusively asymptomatic. ^39^

# Reviewer report: Reviewer response for version 1

**DOI:** 10.5256/f1000research.121958.r135485

**Published:** 2022-05-16

**Authors:** Martha Cecilia Suárez-Mutis

**Affiliations:** 1 Instituto Oswaldo Cruz, Laboratório de Doenças Parasitárias, Fundação Oswaldo Cruz, Rio de Janeiro, Brazil

**Malaria prevalence in Commune 5 in Tumaco (Nariño,** **Colombia)**

 This is a very interesting study about the prevalence of malaria in an urban area of the Pacific Coast of Colombia. The data is relevant, but there are some areas that need further clarification before indexing.

**Introduction**

 Page 3:

 The authors said, *“*To overcome the limitations of optical microscopy and RDTs, molecular detection of malaria parasites through the polymerase chain reaction (PCR) was used”. It is true that optical microscopy and RDT have imitations, but it is important that the authors describe the context in which molecular assays are important.

 The authors said, “Regarding urban malaria, there are challenges in terms of what is meant by “urban”, “peri-urban”, “rural” and “urban malaria”. “Urban malaria” is repeated in this sentence.

 The authors said, “Despite these difficulties, it has been established that urban and peri-urban malaria is caused by population traveling from rural to urban and peri-urban areas ^13^ and trips from rural areas to urban areas and vice versa”. This information is not exact. It depends on the context. Several African and Amazonian cities have larval habitats that determine autochthones malaria cases. On the other hand, what is the difference between “traveling from rural to urban and peri-urban” and “trips from rural areas to urban areas and vice versa”?

 The authors said, “Given the limited research on malaria prevalence carried out in the Colombian territory and the detection of pfhrp 2/3 genes in urban areas, this study was aimed at estimating malaria prevalence, detecting pfhrp2/3 genes, and exploring associated factors in the urban area (Commune 5) in Tumaco during 2019”. From Public Health programs, it is important to detect the percentage of **deletions** of the Pfhrp2/3 genes in a population.

 Page 4:

 About the inclusion criteria, the authors said that “minors who have the authorization of the responsible adult or guardian” were included. Was there not a minimum age for inclusion of children? The authors said that “a prevalence study was conducted in Commune 5 of Tumaco, a malaria-endemic urban area”. The authors should inform the criteria for selection of Commune 5 in Tumaco municipality. Is this the only Commune that has malaria? A better description of Commune 5 is necessary. How many inhabitants live there? Where is Commune 5 located? In the middle or in the periphery of the city? In the figure it seems that there is a river? What is the name of the river? The authors said that “In Tumaco, in the urban area, malaria is concentrated in Commune 5, where nearly 70% of the cases are found and there are breeding sites of Anopheles spp.”. 70% of urban cases are originated of the Commune 5? The idea is not clear. It is much better to say: In the urban area of Tumaco, 70% of malaria cases are found in the Commune 5 where there are breeding sites (our larval habitats) for *Anophelines* spp. too. What kind of larval habitats are in this Commune 5?

 Page 6:

 The authors collected blood for thick smear, RDTs and whole blood and filter paper samples, but in the methods, there is no information about the methods and the quantity of total blood was collected for PCR and filter paper samples. Please add this information.

 On the other hand, for better understanding, we suggest that data about DNA extraction will be added before PCR experiments. The authors only add information about DNA extraction from filter paper but not for the whole blood extraction. Was it made? If whole blood extraction was done, DNA was extracted from what quantity of blood?

 Page 7:

 Please add the reference of the protocol that was used for amplified Pfhrp2/3 genes.

 For entomological collections, how many capture points were sampled? How many days, or how many times were the adult captures done?

 The authors defined “Asymptomatic malaria as Individual with positive microscopy/RDT or PCR, without symptoms”. It is important to remember that with this definition, individuals could be in a prepatogenic period or be an asymptomatic individual. The same reasoning goes for “Submicroscopic malaria”. The individual can be in the prepathogenic period or she/he can be in recovering period. Questions about malaria in the last month or to use malaria drugs in the last month were done?

**Results**:

 Table 3 included some categories that should not be included. For example, the variables “Educational level” and “civil status” for children under 5 years old.

 Page 11:

 Authors said that “and through PCR it was 2.16%”. Exclude “it” in this sentence. Notably, only one individual with symptoms was PCR (+) and 6 with thick smear/RDT were symptomatic. Were these individuals negative in the PCR or was the PCR not done in these patients?

 Authors said that, “The prevalence of amplification of the pfhrp2 gene was 2.16% (95% CI: 1.58% - 2.94%), and for the pfhrp3 gene and the PvSSU gene it was 0”. The literature for *Pfhrp*2/3 usually shows the percentage of gene deletion not the percentage of detection of these genes. It is expected that *P. falciparum* samples carry the gene. It is extremally important that authors confirm if they effectively detected the gene or if they are showing the deletion of the gene. What does the percentage actually mean?

 Page 14:

**Discussion:**

 The authors said that “The prevalence of malaria was 2.97%. It was higher (4.42%) in the 10 to 19 age group”, but in the table 4, the 0-9 age group the prevalence was 4.06. Was there any statistical difference found in malaria cases in children between 0 and 9 years old and 10 and 19 years old?

 The authors said that “The study found malaria in Commune 5, an area believed to have high transmission according to reports from the Vector-Borne Disease Prevention and Control Program in the Nariño Department (API 2014 84.0 cases per 1,000 inhabitants; API 2015 220.5 and API 2016 165.8)”. Please add a reference. A better discussion using previous knowledge of malaria in this area must be added. If this area was classified as high malaria risk in the past, why did the results show this low prevalence? How do we interpret these results? What´s causing the different prevalence between age groups? How do we understand this epidemiology?

 In order to better interpret the results it is necessary to add more information. For example, when was the study done? (Year and month)? In this locality, is malaria seasonal? Was the study carried out during the high malaria season?

 The authors said that “In urban areas, malaria infection is not expected to be prevalent due to better-designed households with better basic utilities, better access to health services, and the absence of mosquito breeding sites”. They use a reference about taxonomy and not specifically urban malaria. Studies about urban malaria around the world show that there are specific determinants for malaria transmission in these areas. Larval habitats usually are very near to these urban areas. This does not seem to be the case in this area where the prevalence was very low.

 The authors found a lack of concordance between microscopy/RDT and PCR tests. As they did not separate microscopy and RDT is difficult to interpret this result. A technical problem can occur when a positive microscopy is comparing with PCR. One question is, did the authors do quality control for thick smears? As primers are very specific, it is difficult to explain a positive thick smear with a negative PCR.

 On the other hand, a positive microscopy with a negative RDT is probably because the limit of detection of RDT. The same rational is true for negative RDT with positive PCR. Cases of negative thick smear with positive RDT could be explained because Pfhrp2 antigen could be detected in the blood until 30 days after a good malaria treatment.

 Anyway, it is necessary that authors add this information in the discussion and include it in the results a separate data for microscopy, RDT and PCR results (including in symptomatic and asymptomatic individuals).

 Page 15:

 The authors said that “In terms of age groups, the highest prevalence of malaria was observed in the 10 to 19 age group and as age increased, prevalence decreased. This result is similar to that reported in other studies. Older people are likely to have a lower risk of becoming ill because they may develop some immunity if they have been previously exposed to the infection”. Was there any question about malaria antecedents in the questionnaire? How many malaria episodes did the participants have before the study? Or, how many participants had at least one previous malaria episode before the study? These are important questions that the authors did not address.

 The authors said that “Furthermore, asymptomatic malaria may precede symptomatic disease, and may confer partial immunity against infection”. Asymptomatic *Plasmodium *infection is an important field of study and until now many questions continue open for discussion. It seems clear that asymptomatic infections occur in individuals with several past malaria episodes and this status is product of clinical immunity develop along time. This “status of *Plasmodium* asymptomatic infection” is different of those individuals whose blood were collected during the pre-pathogenic period before the beginning of malaria paroxysm. They could be in the “incubation period” and the number of parasites circulating in blood do not still reach the parasitic threshold for clinical symptoms. A better discussion about authors results and their interpretation must be added.

 About discussion of Pfhrp2, we added above this sentence: “Authors said that “The prevalence of amplification of the pfhrp2 gene was 2.16% (95% CI: 1.58% - 2.94%), and for the pfhrp3 gene and the PvSSU gene it was 0”. The literature for *Pfhrp*2/3 usually show the percentage of gene deletion not the percentage of detection of these genes. It is expected that *P falciparum* samples carry the gene. It is extremally important that authors confirm if they effectively detected the gene or if they are showing the deletion of the gene. What does the percentage actually mean?”. For the discussion, it is very important to know what was really detected by the authors - the deletion of gene or the gene?

 The authors said that “On the other hand, in the Commune 5 neighborhoods of Tumaco, *An. albimanus* was found in artificial ponds and wells”. This data was not added in the results section.

 Regarding anopheline transmission in this study, it would be interesting if the authors added a discussion about ecological conditions for malaria transmission of *An. albimanus.* Furthermore, a critical analysis of the constraints of entomological studies must be incorporated.

Is the work clearly and accurately presented and does it cite the current literature?

Yes

If applicable, is the statistical analysis and its interpretation appropriate?

Partly

Are all the source data underlying the results available to ensure full reproducibility?

Yes

Is the study design appropriate and is the work technically sound?

Partly

Are the conclusions drawn adequately supported by the results?

Partly

Are sufficient details of methods and analysis provided to allow replication by others?

Yes

Reviewer Expertise:

Malaria epidemiology and molecular epidemiology of Plasmodium infections; malaria diagnosis, Pfhrp2/3 deletion detection; asymptomatic Plasmodium infections.

I confirm that I have read this submission and believe that I have an appropriate level of expertise to confirm that it is of an acceptable scientific standard, however I have significant reservations, as outlined above.

# Response

**Published:** 2022-08-31

**Authors:** Pablo Enrique Chaparro Narváez

**Affiliations:** Instituto Nacional de Salud (Colombia), Colombia

Thank you for your constructive review. These are our answers for your review:

**Introduction**

 Page 3:

 The authors said, *“*To overcome the limitations of optical microscopy and RDTs, molecular detection of malaria parasites through the polymerase chain reaction (PCR) was used”. It is true that optical microscopy and RDT have imitations, but it is important that the authors describe the context in which molecular assays are important.

1. The following paragraph was included: “PCR is a more sensitive diagnostic method to detect asymptomatic infections with very low parasite density. It may be useful in studies of submicroscopic infections, although its utility depends on the epidemiological importance of low-density infections, which have not yet been characterized” (Framework for malaria elimination)

The authors said, “Regarding urban malaria, there are challenges in terms of what is meant by “urban”, “peri-urban”, “rural” and “urban malaria”. “Urban malaria” is repeated in this sentence.

1. We corrected

The authors said,

 “Despite these difficulties, it has been established that urban and peri-urban malaria is caused by population traveling from rural to urban and peri-urban areas13 and trips from rural areas to urban areas and vice versa”. This information is not exact. It depends on the context. Several African and Amazonian cities have larval habitats that determine autochthones malaria cases. On the other hand, what is the difference between “traveling from rural to urban and peri-urban” and “trips from rural areas to urban areas and vice versa”?

1. We adjusted: “Despite these difficulties, it has been established that urban and peri-urban malaria may be transmitted by population traveling from rural to urban and peri-urban areas (14,15) Local transmission has also been suggested in urban areas where the presence of Anopheles, as a competent vector, and infected people converge (13)”

The authors said, “Given the limited research on malaria prevalence carried out in the Colombian territory and the detection of pfhrp 2/3 genes in urban areas, this study was aimed at estimating malaria prevalence, detecting pfhrp2/3 genes, and exploring associated factors in the urban area

 (Commune 5) in Tumaco during 2019”. From Public Health programs, it is important to detect the percentage of **deletions **of the Pfhrp2/3 genes in a population.

1. One of the objectives of the study was to detect gene fragments of Pfhrp2/3. The deletion of these genes was not evaluated. However, to improve malaria elimination programs, it is necessary to determine these deletions since, at least in Colombia, the RDTs used for diagnosis are based on these genes, and of course, deletions would generate false negatives.

**Methods**

 Page 4:

 About the inclusion criteria, the authors said that “minors who have the authorization of the responsible adult or guardian” were included. Was there not a minimum age for inclusion of children?

1. There is no minimum age for the inclusion of children.

The authors said that “a prevalence study was conducted in Commune 5 of Tumaco, a malaria-endemic urban area”. The authors should inform the criteria for selection of Commune 5 in Tumaco municipality. Is this the only Commune that has malaria?

1. We corrected: In the urban area of Tumaco, all five communes are endemic to malaria. In this city, 70% of malaria cases are reported from commune 5 in the last ten years

A better description of Commune 5 is necessary. How many inhabitants live there? Where is Commune 5 located? In the middle or in the periphery of the city? In the figure it seems that there is a river? What is the name of the river?

1. We corrected: Commune 5, with 26,000 inhabitants, is in the continental area of Tumaco on the low-tide land and nearby estuaries. The presence of Anophelines sppn breeding sites (ponds, puddles, and lakes for shrimp farming) and the high density of the population make this commune of high risk of malaria transmission.

In the figure, the image corresponds to an estuary. It's not a river.

 The authors said that “In Tumaco, in the urban area, malaria is concentrated in Commune 5, where nearly 70% of the cases are found and there are breeding sites of Anopheles spp.”. 70% of urban cases are originated of the Commune 5? The idea is not clear. It is much better to say: In the urban area of Tumaco, 70% of malaria cases are found in the Commune 5 where there are breeding sites (our larval habitats) for *Anophelines *spp. too. What kind of larval habitats are in this Commune 5?

1. We included the suggested phrase and larval habitats.

Page 6:

 The authors collected blood for thick smear, RDTs and whole blood and filter paper samples, but

 in the methods, there is no information about the methods and the quantity of total blood was collected for PCR and filter paper samples. Please add this information.

1. We have included: For PCR, two drops of blood were obtained from the same finger puncture and were then put on the filter paper

On the other hand, for better understanding, we suggest that data about DNA extraction will be added before PCR experiments.

1. We have made the suggested change.

The authors only add information about DNA extraction from filter paper but not for the whole blood extraction. Was it made? If whole blood extraction was done, DNA was extracted from what quantity of blood?

1. We have included: 10 mm² of Whatman #3 filter paper impregnated with blood were taken and genomic DNA extraction was performed according to the protocol described in the article. This procedure was performed in duplicate.

Page 7:

 Please add the reference of the protocol that was used for amplified Pfhrp2/3 genes.

1. We have included: For the amplification of the different fragments of the genes mentioned, we designed the specific primers and the PCR conditions for each of them were standardized. For the design of the primers, the GC content, dimer formation, loop formation, palindromes and Tm were taken into account. The sequences of the HRP2/HRP3 and PvSSU genes published in the Genbank were used, an alignment was performed using clustalW (Refseq) and the primers were designed from conserved regions of the sequences obtained.

For entomological collections, how many capture points were sampled? How many days, or how many times were the adult captures done?

1. We have included: Between October 1 and 23, 2019, 81 capture points were sampled in 25 homes and 83 breeding places.

The authors defined “Asymptomatic malaria as Individual with positive microscopy/RDT or PCR, without symptoms”. It is important to remember that with this definition, individuals could be in a

 prepatogenic period or be an asymptomatic individual. The same reasoning goes for “Submicroscopic malaria”. The individual can be in the prepathogenic period or she/he can be in recovering period. Questions about malaria in the last month or to use malaria drugs in the last month were done?

1. In the applied questionnaire were included the questions:

- Have you had malaria in the last year, apart from the current episode?
- When was the last time you had malaria?
- Have you taken antimalarial drugs in the last 15 days?        

**Results**:

 Table 3 included some categories that should not be included. For example, the variables

 “Educational level” and “civil status” for children under 5 years old.

  

1. We corrected:

Table 3. Characteristics of the Surveyed Population. Tumaco, July to December 2019

**Characteristics**

**Total**

**Unweighted count**

**Weighted count**

**n**

**%**

**95% Confidence Interval**

**Age groups**

 0-9

 272

 18,32

 16,0

 20,9

 10-19

 318

 21,47

 19,1

 24,1

 20-49

 626

 40,88

 38,64

 43,15

 ≥50

 288

 19,33

 16,94

 21,98

**Sex**

 Male

 531

 35,56

 33,5

 37,7

 Female

 973

 64,44

 62,3

 66,5

**Ethnic group**

 Black, African descent

 1.439

 95,23

 91,4

 97,4

 Others

 65

 4,77

 2,6

 8,6

**Years living in the house**

 0 to 4

 373

 25,60

 22,5

 28,9

 5 to 9

 397

 25,97

 22,4

 29,9

 10 or more

 714

 47,03

 42,6

 51,5

 No information

 20

 1,40

 0,8

 2,3

**Educational level** *

 Preschool, primary

 555

 40,75

 37,05

 44,55

 High school

 628

 45,79

 42,88

 48,73

 University

 113

 8,54

 6,43

 11,24

 None

 72

 4,93

 3,57

 6,77

**Civil status** **

 Has a partner

 585

 52.78

 49.08

 56.45

 No partner

 526

 47.22

 43.55

 50.92

**Occupation**

 Worker

 450

 30,04

 26,4

 34,0

 Housework

 399

 26,26

 24,0

 28,7

 Early childhood

 158

 10,46

 8,8

 12,4

 Childhood

 178

 12,26

 10,7

 14,0

 Adolescence

 218

 14,49

 12,7

 16,5

 Unemployed

 36

 2,29

 1,6

 3,2

 Pensioner

 14

 0,86

 0,5

 1,4

 Other

 51

 3,34

 2,4

 4,6

**Family income**

 <1 MLW (Monthly Legal Wage)

 1.219

 79,37

 72,8

 84,7

 1 or more MLW

 281

 20,33

 15,1

 26,8

 Does not know

 4

 0,30

 0,1

 1,7

**Malaria in the last year**

 10

 0,67

 0,32

 1,40

 * In the “Educational level” variable, 136 children under 5 years of age were excluded.

 ** In the “Civil status” variable, 393 children under 14 years of age were excluded.

 Page 11:

 Authors said that “and through PCR it was 2.16%”. Exclude “it” in this sentence.

1. We exclude “it” in this sentence.

Notably, only one individual with symptoms was PCR (+) and 6 with thick smear/RDT were symptomatic. Were these individuals negative in the PCR or was the PCR not done in these patients?

1. Yes, these individuals were negative in the PCR. All these persons were examined by PCR.

Authors said that, “The prevalence of amplification of the pfhrp2 gene was 2.16% (95% CI: 1.58% -

 2.94%), and for the pfhrp3 gene and the PvSSU gene it was 0”. The literature for *Pfhrp*2/3 usually shows the percentage of gene deletion not the percentage of detection of these genes. It is

 expected that *P. falciparum *samples carry the gene. It is extremally important that authors confirm if they effectively detected the gene or if they are showing the deletion of the gene. What does the percentage actually mean?

1. Fragments of the Pfhrp2/3 genes were detected by PCR amplification. No assays were performed to assess the deletion of these genes. The percentage shown corresponds to the percentage of amplification of the Pfhrp2/3 genes.

**Discussion:**

 Page 14:

 The authors said that “The prevalence of malaria was 2.97%. It was higher (4.42%) in the 10 to 19 age group”, but in the table 4, the 0-9 age group the prevalence was 4.06. Was there any statistical difference found in malaria cases in children between 0 and 9 years old and 10 and 19 years old?

1. There were no statistically significant differences (p: 0.83). We adjust: It was higher in the 10 to 19 (4.42%) and 0 to 9 (4.06%) age groups

The authors said that “The study found malaria in Commune 5, an area believed to have high transmission according to reports from the Vector-Borne Disease Prevention and Control Program

 in the Nariño Department (API 2014 84.0 cases per 1,000 inhabitants; API 2015 220.5 and API 2016 165.8)”. Please add a reference.

1. We add: Pilar Perez, Vector-Borne Disease Prevention and Control Program in the Nariño Department, personal communication

A better discussion using previous knowledge of malaria in this area must be added. If this area was classified as high malaria risk in the past, why did the results show this low prevalence? How do we interpret these results? What´s causing the different prevalence between age groups? How do we understand this epidemiology?

  

1. We add: The decrease in the prevalence of malaria over time is possibly due to the intensification of actions to search for and eliminate breeding sites, massive use of mosquito nets, and social participation as measures implemented for Commune 5. Diagnosis and treatment actions also have contributed, despite the difficulties that exist in accessing services. In this way, it has reduced human contact (infected) —a vector that has contributed to the decline in the burden of this disease.

The higher prevalence of malaria in people under 20 years of age suggests that the infection may have been acquired locally. Unlike Africa, which has a high transmission and where the population, most affected in terms of morbidity and mortality is under 5 years of age, in areas of low transmission such as Colombia, malaria occurs in older age groups. Among the aspects that are considered to think that the transmission occurs in the urban area are confirming the infection in the residents of these areas; evidence of the presence of adult Anopheles that are biting the inhabitants of urban dwellings and identifying breeding sites near them (40).

 In order to better interpret the results it is necessary to add more information. For example, when was the study done? (Year and month)? In this locality, is malaria seasonal? Was the study carried out during the high malaria season?

1. The blood samples were taken during the dry season, between July and September 2019. Malaria in the urban area of Tumaco has not shown a clear seasonal behavior.

The authors said that “In urban areas, malaria infection is not expected to be prevalent due to better-designed households with better basic utilities, better access to health services, and the absence of mosquito breeding sites”. They use a reference about taxonomy and not specifically urban malaria. Studies about urban malaria around the world show that there are specific determinants for malaria transmission in these areas. Larval habitats usually are very near to these urban areas. This does not seem to be the case in this area where the prevalence was very low.

1. We change the reference: Larson P, Eisenberg J, Berrocal V, Mathanga D, Wilson M. An urban-to-rural continuum of malaria risk: new analytic approaches characterize patterns in Malawi. Malaria Journal 20, 418 (2021) (ref 46)

The authors found a lack of concordance between microscopy/RDT and PCR tests. As they did not separate microscopy and RDT is difficult to interpret this result. A technical problem can occur when a positive microscopy is comparing with PCR. One question is, did the authors do quality control for thick smears? As primers are very specific, it is difficult to explain a positive thick smear with a negative PCR.

1. The National Institute of Health performed quality control on 10% (153) of the samples analyzed by optical microscopy in accordance with national recommendations.

On the other hand, a positive microscopy with a negative RDT is probably because the limit of detection of RDT. The same rational is true for negative RDT with positive PCR. Cases of negative thick smear with positive RDT could be explained because Pfhrp2 antigen could be detected in the blood until 30 days after a good malaria treatment.

 Anyway, it is necessary that authors add this information in the discussion and include it in the results a separate data for microscopy, RDT and PCR results (including in symptomatic and asymptomatic individuals).

1. We add: “On the other hand, positive microscopy with a negative RDT is probably because of the limit of detection of RDT. The same rationale is true for negative RDT with positive PCR. Cases of negative thick smear with positive RDT could be explained because Pfhrp2 antigen could be detected in the blood until 30 days after a good malaria treatment.”

We include in the results:

 Table 5. Test results according to symptoms. Unweighted count

 Commune 5 neighborhoods, Tumaco (Nariño, Colombia). October 2019

 Test

 Symptoms

 Total

 Yes

 No

 Optical microscopy (RDT)

 6 (5)

 5 (3)

 11 (8)

 PCR

 1

 27

 28

 Total

 7

 33

 39

 In parentheses, the cases diagnosed with RDT

 Page 15:

 The authors said that “In terms of age groups, the highest prevalence of malaria was observed in the 10 to 19 age group and as age increased, prevalence decreased. This result is similar to that reported in other studies. Older people are likely to have a lower risk of becoming ill because they may develop some immunity if they have been previously exposed to the infection”. Was there any question about malaria antecedents in the questionnaire? How many malaria episodes did the participants have before the study? Or, how many participants had at least one previous malaria episode before the study? These are important questions that the authors did not address.

1.  We included in the study the questions
  - Have you had malaria in the last year, apart from the current episode?
  - When was the last time you had malaria?
  - Have you taken antimalarial drugs in the last 15 days? 

The results are presented in the corresponding section as follows:

 Ten individuals had malaria in the last year. Of them, none had malaria in the last month. They also did not have positive test results for malaria in the last 15 days, and they had not consumed antimalarial drugs.

 Less than 1% of participants had malaria in the last year and none in the last month. It is possible that in areas of low endemicity, such as Colombia, the lowest prevalence in older age groups is due to the fact that the population that may have less exposure was interviewed. For example, women who remain at home dedicated to household chores and individuals of school age who go to school.

 The authors said that “Furthermore, asymptomatic malaria may precede symptomatic disease, and may confer partial immunity against infection”. Asymptomatic *Plasmodium *infection is an important field of study and until now many questions continue open for discussion. It seems clear that asymptomatic infections occur in individuals with several past malaria episodes and this status is product of clinical immunity develop along time. This “status of *Plasmodium *asymptomatic infection” is different of those individuals whose blood were collected during the pre-pathogenic period before the beginning of malaria paroxysm. They could be in the “incubation period” and the number of parasites circulating in blood do not still reach the parasitic threshold for clinical symptoms. A better discussion about authors results and their interpretation must be added.

  

1. In this study, participants were sampled at a single point in time. Those who were classified as asymptomatic had no history of illness in the last month and had not used antimalarial drugs in the last 15 days. For them, it is difficult to establish if they were in the incubation period of the disease or if they were carriers of a low number of parasites. Resolving a situation like this requires follow-up. In this regard, a study of 268 people followed for 29 months in western Kenya, in a high-transmission area, discovered that asymptomatic infections were highly likely to be followed by symptomatic disease (ref 55). However, this does not seem to be the case in Commune 5 of Tumaco, which has a low transmission and where complicated malaria and malaria mortality do not occur.

About discussion of Pfhrp2, we added above this sentence: “Authors said that “The prevalence of amplification of the pfhrp2 gene was 2.16% (95% CI: 1.58% - 2.94%), and for the pfhrp3 gene and the PvSSU gene it was 0”. The literature for *Pfhrp*2/3 usually show the percentage of gene deletion not the percentage of detection of these genes. It is expected that *P falciparum *samples carry the gene. It is extremally important that authors confirm if they effectively detected the gene or if they are showing the deletion of the gene. What does the percentage actually mean?”. For the discussion, it is very important to know what was really detected by the authors - the deletion of gene or the gene?

1. We included: In the context of our study due to insufficient resources, we did not evaluate the deletion of these genes, we only amplified a fragment of these genes by means of PCR. However, in order to improve the management of clinical cases and design strategies for diagnosis, the detection of deletions in the HRP2/3 genes is relevant, since, as has been described, these can affect the accuracy of PDR in malaria endemic regions and of course in malaria control programs.

The authors said that “On the other hand, in the Commune 5 neighborhoods of Tumaco, *An.*

*albimanus *was found in artificial ponds and wells”. This data was not added in the results section.

1. These data is in the results section: “83 breeding sites were found. Out of those, two had Anopheles mosquito larvae, one in the Unión Victoria neighborhood and the other one in the Obrero neighborhood. One breeding site was a pond and the other one was a well. 32 larvae were collected in both breeding sites. 27 of them were in late stages (3rd and 4th) and were identified as *An. albimanus*.”

Regarding anopheline transmission in this study, it would be interesting if the authors added a discussion about ecological conditions for malaria transmission of *An. albimanus. *

1. *An albimanus* is considered a primary malaria vector in Colombia and particularly in Tumaco (ref 59). The presence of *An. albimanus* in different neighborhoods of Commune 5 provides partial evidence of the possible local transmission of malaria. This mosquito has been considered an opportunistic species due to its ability to adapt to different breeding sites (ref 60). Despite the small number of adults collected, there is a risk of transmission, taking into account that the maximum biting activity occurs in the peridomicile between 18:00 and 19:00 hours.

Furthermore, a critical analysis of the constraints of entomological studies must be incorporated.

1. Particular attention requires the limitation related to entomological aspects. The results presented are only an approximation to the subject. The difficulties presented did not allow them an adequate study of the ecology of the vector and to characterize the aspects related to the vector's biting density, feeding patterns, biting activity, parity rates, and resting behavior, among other issues that represent important factors for malaria transmission within the Commune 5.
